# Cost-Effectiveness of Gene-Specific Prevention Strategies for Ovarian and Breast Cancer

**DOI:** 10.1001/jamanetworkopen.2023.55324

**Published:** 2024-02-09

**Authors:** Xia Wei, Li Sun, Eric Slade, Caitlin T. Fierheller, Samuel Oxley, Ashwin Kalra, Jacqueline Sia, Michail Sideris, W. Glenn McCluggage, Nathan Bromham, Katharina Dworzynski, Adam N. Rosenthal, Adam Brentnall, Stephen Duffy, D. Gareth Evans, Li Yang, Rosa Legood, Ranjit Manchanda

**Affiliations:** 1 Department of Health Services Research and Policy, London School of Hygiene & Tropical Medicine, London, United Kingdom; 2 Wolfson Institute of Population Health, Queen Mary University of London, London, United Kingdom; 3 National Institute for Health and Care Excellence, London, United Kingdom; 4 Department of Gynaecological Oncology, Barts Health NHS Trust, Royal London Hospital, London, United Kingdom; 5 Department of Pathology, Belfast Health & Social Care Trust, Royal Victoria Hospital, Belfast, United Kingdom; 6 Department of Gynaecology, University College London Hospitals NHS Foundation trust, London, United Kingdom; 7 Department of Women’s Cancer, UCL EGA Institute for Women’s Health, University College London, London, United Kingdom; 8 Manchester Centre for Genomic Medicine, Division of Evolution, Infection and Genomic Sciences, University of Manchester, MAHSC, Manchester, United Kingdom; 9 School of Public Health, Peking University, Beijing, China; 10 MRC Clinical Trials Unit at UCL, Institute of Clinical Trials & Methodology, Faculty of Population Health Sciences, University College London, London, United Kingdom

## Abstract

**Importance:**

Pathogenic variants (PVs) in *BRCA1*,* BRCA2*,* PALB2*,* RAD51C*,* RAD51D*, and* BRIP1* cancer susceptibility genes (CSGs) confer an increased ovarian cancer (OC) risk, with *BRCA1*,* BRCA2*,* PALB2*,* RAD51C*, and* RAD51D* PVs also conferring an elevated breast cancer (BC) risk. Risk-reducing surgery, medical prevention, and BC surveillance offer the opportunity to prevent cancers and deaths, but their cost-effectiveness for individual CSGs remains poorly addressed.

**Objective:**

To estimate the cost-effectiveness of prevention strategies for OC and BC among individuals carrying PVs in the previously listed CSGs.

**Design, Setting, and Participants:**

In this economic evaluation, a decision-analytic Markov model evaluated the cost-effectiveness of risk-reducing salpingo-oophorectomy (RRSO) and, where relevant, risk-reducing mastectomy (RRM) compared with nonsurgical interventions (including BC surveillance and medical prevention for increased BC risk) from December 1, 2022, to August 31, 2023. The analysis took a UK payer perspective with a lifetime horizon. The simulated cohort consisted of women aged 30 years who carried *BRCA1*,* BRCA2*,* PALB2*,* RAD51C*,* RAD51D*, or *BRIP1* PVs. Appropriate sensitivity and scenario analyses were performed.

**Exposures:**

CSG-specific interventions, including RRSO at age 35 to 50 years with or without BC surveillance and medical prevention (ie, tamoxifen or anastrozole) from age 30 or 40 years, RRM at age 30 to 40 years, both RRSO and RRM, BC surveillance and medical prevention, or no intervention.

**Main Outcomes and Measures:**

The incremental cost-effectiveness ratio (ICER) was calculated as incremental cost per quality-adjusted life-year (QALY) gained. OC and BC cases and deaths were estimated.

**Results:**

In the simulated cohort of women aged 30 years with no cancer, undergoing both RRSO and RRM was most cost-effective for individuals carrying *BRCA1* (RRM at age 30 years; RRSO at age 35 years), *BRCA2* (RRM at age 35 years; RRSO at age 40 years), and *PALB2* (RRM at age 40 years; RRSO at age 45 years) PVs. The corresponding ICERs were −£1942/QALY (−$2680/QALY), −£89/QALY (−$123/QALY), and £2381/QALY ($3286/QALY), respectively. RRSO at age 45 years was cost-effective for *RAD51C*, *RAD51D*, and *BRIP1* PV carriers compared with nonsurgical strategies. The corresponding ICERs were £962/QALY ($1328/QALY), £771/QALY ($1064/QALY), and £2355/QALY ($3250/QALY), respectively. The most cost-effective preventive strategy per 1000 PV carriers could prevent 923 OC and BC cases and 302 deaths among those carrying *BRCA1*; 686 OC and BC cases and 170 deaths for *BRCA2*; 464 OC and BC cases and 130 deaths for *PALB2*; 102 OC cases and 64 deaths for *RAD51C*; 118 OC cases and 76 deaths for *RAD51D*; and 55 OC cases and 37 deaths for *BRIP1*. Probabilistic sensitivity analysis indicated both RRSO and RRM were most cost-effective in 96.5%, 89.2%, and 84.8% of simulations for *BRCA1*, *BRCA2*, and* PALB2 *PVs, respectively, while RRSO was cost-effective in approximately 100% of simulations for *RAD51C*,* RAD51D*, and *BRIP1 *PVs.

**Conclusions and Relevance:**

In this cost-effectiveness study, RRSO with or without RRM at varying optimal ages was cost-effective compared with nonsurgical strategies for individuals who carried *BRCA1*, *BRCA2*, *PALB2*, *RAD51C*,* RAD51D*, or *BRIP1* PVs. These findings support personalizing risk-reducing surgery and guideline recommendations for individual CSG-specific OC and BC risk management.

## Key Points

**Question:**

How cost-effective are ovarian and breast cancer risk reduction strategies among women with pathogenic variants in individual cancer susceptibility genes (ie, *BRCA1*, *BRCA2*,* PALB2*, *RAD51C*, *RAD51D*, and *BRIP1*)?

**Findings:**

This economic evaluation using a decision-analytic Markov model with a simulated cohort of women aged 30 years found that undergoing both risk-reducing mastectomy (RRM) and risk-reducing salpingo-oophorectomy (RRSO) was most cost-effective, maximizing cancers prevented for individuals carrying *BRCA1* (RRM at age 30 years; RRSO at age 35 years), *BRCA2* (RRM at age 35 years; RRSO at age 40 years), and *PALB2* (RRM at age 40 years; RRSO at age 45 years) pathogenic variants, while RRSO was cost-effective at age 45 years for women with *RAD51C*, *RAD51D*, and *BRIP1* pathogenic variants.

**Meaning:**

These findings support personalizing risk-reducing surgery and guideline recommendations for individual cancer susceptibility gene–specific ovarian and breast cancer risk management.

## Teaser

This economic evaluation estimates the cost-effectiveness of prevention strategies for ovarian and breast cancer among individuals with pathogenic variants in cancer susceptibility genes *BRCA1*,* BRCA2*,* PALB2*,* RAD51C*,* RAD51D*, and* BRIP1*.

## Introduction

Approximately 15% to 20% of ovarian cancer (OC) cases are caused by pathogenic variants (PVs) in cancer susceptibility genes (CSGs) including *BRCA1 *(OMIM 113705), *BRCA2 *(OMIM 600185), *PALB2 *(OMIM 610355), *RAD51C *(OMIM 602774), *RAD51D *(OMIM 602954), and *BRIP1 *(OMIM 605882),^1,2^ which confer varying lifetime OC risks of 44% to 48%, 17% to 20%, 5%, 11%, 13%, and 6%, respectively.^3,4,5,6,7^ *BRCA1*, *BRCA2*, *PALB2*,* RAD51C*, and* RAD51D* PVs also confer elevated lifetime breast cancer (BC) risks of 65% to 72%, 61% to 69%, 53%, 21%, and 20%, respectively.^3,4,5,6^ Increasing awareness and acceptability of genetic testing and falling costs, coupled with changing clinical practices, including increasing genetic testing at cancer diagnosis^1,8,9^ and recent calls for population testing,^10,11,12,13^ are leading to ever-increasing identification of women with PVs in moderate- or high-penetrance OC or BC CSGs.

Recommendations for unaffected women with increased OC or BC risk include surveillance, medical prevention, and risk-reducing surgery.^14,15,16,17^ Enhanced BC surveillance (depending on age and risk category) is recommended by the National Institute for Health and Care Excellence (NICE) familial BC guideline for individuals carrying *BRCA1*, *BRCA2*, or *PALB2* PVs and women with a 17% to 30% (ie, moderate) lifetime BC risk,^14^ but OC surveillance is unavailable given lack of survival or mortality benefit.^18,19^ Medical prevention with tamoxifen or anastrozole reduces premenopausal or postmenopausal BC risk, respectively.^20,21^ Risk-reducing surgery, including risk-reducing salpingo-oophorectomy (RRSO) and risk-reducing mastectomy (RRM), remain the most clinically effective preventive strategies and are increasingly undertaken.^22,23,24^

Although recent guidelines have started to incorporate recommendations for individuals who carry PVs in non-*BRCA* CSGs,^15,17,25^ existing guidelines have mainly focused on *BRCA1 *and *BRCA2*, and the optimal timing of management for all CSGs is inadequately addressed. Available modeling studies show RRSO and/or RRM are cost-effective compared with BC surveillance or no surgery for *BRCA1 *and *BRCA2*, while the ages at surgery varied.^26,27,28^ To our knowledge, no cost-effectiveness studies for non-*BRCA* CSG PV carriers (ie, *PALB2*, *RAD51C*, *RAD51D*, or *BRIP1*) have been undertaken.^26,27,28^ Additionally, UK health system RRSO and RRM–specific cost-effectiveness data are lacking for *BRCA1 *and* BRCA2*. Given the variation in CSG-associated cancer risks, diversity in health care systems, model structures and assumptions, these knowledge gaps need addressing.

This study evaluates the cost-effectiveness of eligible prevention and surveillance strategies and the optimal timing of management in individuals carrying *BRCA1*, *BRCA2*, *PALB2*, *RAD51C*, *RAD51D*, or* BRIP1* PVs. This analysis was used to inform the UK NICE guideline on identifying and managing familial and genetic OC risk and was presented in the (closed) Guideline Committee Meeting.

## Methods

This study followed the Consolidated Health Economic Evaluation Reporting Standards (CHEERS)^29^ reporting guideline and NICE health technology evaluations manual.^30^ It received ethics approval from the London School of Hygiene & Tropical Medicine Ethics Committee.

### Model Overview

**Figure 1.**
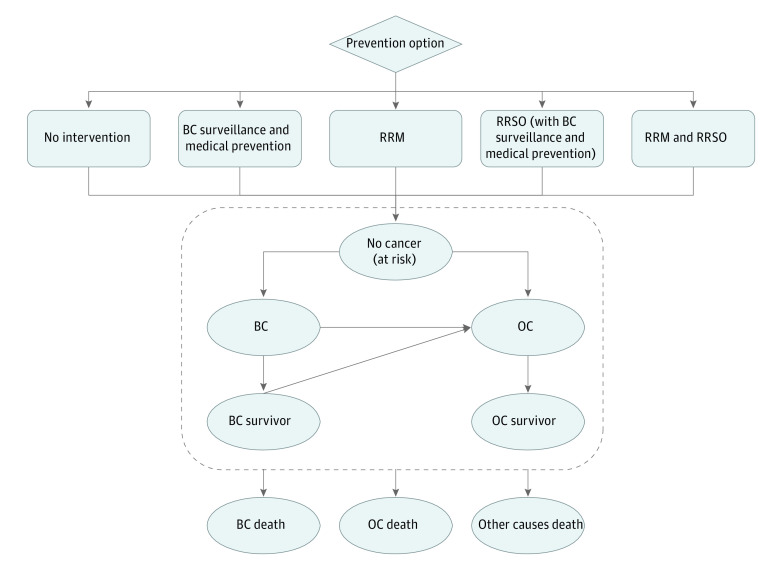
Model Overview The figure includes the decision tree pathway for choosing eligible surveillance and prevention strategies and the schematic illustration of the health states and key transitions for the Markov model. Model structure and compared strategies are adjusted based on the ovarian cancer (OC) and breast cancer (BC) risk level conferred by pathogenic variants in individual cancer susceptibility genes, ie, *BRCA1*, *BRCA2*, *PALB2*, *RAD51C*, *RAD51D*, and *BRIP1*. RRM indicates risk-reducing mastectomy; and RRSO, risk-reducing salpingo-oophorectomy.

We developed a decision-analytic Markov model (Figure 1) using TreeAge Pro 2021 (TreeAge) to evaluate the costs and outcomes of OC and BC prevention and BC surveillance strategies for healthy women with *BRCA1*, *BRCA2*, *PALB2*, *RAD51C*, *RAD51D*, or *BRIP1* PVs. The target population began in a healthy (ie, no cancer) state and progressed through health states including OC, BC, OC survivor, BC survivor, cancer-specific death, or all-cause death. We adjusted costs and utilities for BC and OC by stage distribution and PV carrier status (where relevant) (eMethods 2 and 4 in Supplement 1).^18,31^ OC and BC diagnoses were assumed to be independent, and the probability of developing OC after BC was included, but BC after OC was excluded given worse OC survival and the rare possibility of this occurring.^32^ The model simulation started at age 30 years (given ages of cancer risk onset and availability of risk management options) and cycled annually until age 100 years. The main model structure was the backbone on which we overlaid the CSG-specific characteristics and strategies based on OC and BC risks conferred by them (Table 1).

**Table 1.** Cancer Risk Level and Management Strategies by CSG

CSG	Lifetime OC risk	Lifetime BC risk	Source	Base case management strategies^a^
*BRCA1*	44%-48%	65%-72%	Chen at al,^3^ 2020, and Kuchenbaecker et al,^4^ 2017	High-risk BC surveillance and tamoxifen from age 30 y RRM at age 30 y RRSO at age 35 y with high-risk BC surveillance and tamoxifen from age 30 y RRM at age 30 and RRSO at age 35 y
*BRCA2*	17%-20%	61%-69%	Chen at al,^3^ 2020, and Kuchenbaecker et al,^4^ 2017	High-risk BC surveillance and tamoxifen from age 30 y RRM at age 35 y RRSO at age 40 y with high-risk BC surveillance and tamoxifen from age 30 y RRM at age 35 y and RRSO at age 40 y
*PALB2*	5%	53%	Yang et al,^5^ 2020	High-risk BC surveillance and tamoxifen from age 30 y RRM at age 40 y RRSO at age 45 y with high-risk BC surveillance and tamoxifen from age 30 y RRM at age 40 y and RRSO at age 45 y
*RAD51C*	11%	21%	Yang et al,^6^ 2020	Moderate-risk BC surveillance and tamoxifen from age 40 y RRSO at age 45 y with moderate-risk BC surveillance and tamoxifen from age 40 y
*RAD51D*	13%	20%	Yang et al,^6^ 2020	Moderate-risk BC surveillance and tamoxifen from age 40 y RRSO at age 45 y with moderate-risk BC surveillance and tamoxifen from age 40 y
*BRIP1*	5.8%	Not increased	Ramus et al,^7^ 2015	No surgery RRSO at age 45 y

Abbreviations: BC, breast cancer; CSG, cancer susceptibility gene; OC, ovarian cancer; RRM, risk-reducing mastectomy; RRSO, risk-reducing salpingo-oophorectomy.

^a^ Uptake of risk-reducing surgery and BC surveillance was assumed as 100%. High-risk BC surveillance refers to annual magnetic resonance imaging from age 30 to 49 years and annual mammography from age 40 to 69 years for *BRCA1*, *BRCA2*, *PALB2* pathogenic variant carriers based on National Institute of Health and Care Excellence familial BC guideline^14^; moderate-risk BC surveillance refers to annual mammography from age 40 to 59 years and routine triennial mammography per national screening program from age 60 years for women with 17% to 30% lifetime BC risk based on National Institute of Health and Care Excellence familial BC guideline.^14^ An uptake rate of 16.3% was applied for medical prevention, and the duration of medical prevention was assumed to be 5 years.^33^

Individuals carrying *BRCA1*, *BRCA2*, or *PALB2* PVs have increased OC risk and high BC risk. They receive high-risk BC surveillance per NICE familial BC guidelines^14^: annual magnetic resonance imaging (age 30-49 years) and annual mammography (age 40-69 years). Medical prevention with tamoxifen (premenopausally) or anastrozole (postmenopausally) reduces BC risk by approximately 30% or 50%, respectively.^20,21^ The reference strategy for *BRCA1*, *BRCA2*, and *PALB2* PV carriers was therefore BC surveillance coupled with medical prevention. We assumed a conservative 16.3% medical prevention uptake from ages 30 to 35 years.^33^ Eligible surgical prevention strategies included undergoing RRM, RRSO, or both (RRSO plus RRM). In line with clinical and practice guidelines,^14,15,16,17^ given differences in CSG-specific ages of cancer onset,^4,5^ age of RRM was assumed at 30, 35, and 40 years and age of RRSO at 35, 40, and 45 years for *BRCA1*, *BRCA2*, and *PALB2* PV carriers, respectively. Women undergoing RRSO (alone) also undergo BC surveillance and medical prevention given increased BC risk. Women undergoing RRM do not receive BC surveillance or medical prevention after surgery. We assumed that 80% of premenopausal women would receive hormone therapy (HT) after RRSO until the average menopause age (51 years).^34^ Excess risk of coronary heart disease (CHD) after premenopausal RRSO was modeled, including a 3% increase in risk of mortality without HT.^35,36^ Per a recent meta-analysis,^37^ we assumed premenopausal RRSO only reduced BC risk for *BRCA2 *and* PALB2* PV carriers (not *BRCA1 *PV carriers). OC surveillance was not incorporated given lack of survival and mortality benefit.^18,19^

Individuals who carry *RAD51C *or* RAD51D* PVs have increased OC risk and moderate BC risk. They undergo RRSO at age 45 years (base case) and receive NICE-recommended moderate-risk BC management^14^: annual mammography (age 40-59 years) and thereafter routine triennial mammography (general population screening program) and medical prevention (age 40-45 years). The reference strategy consisted of BC surveillance and medical prevention. *RAD51C *and *RAD51D* PV carriers do not undergo RRM, as BC risk is less than the threshold (30%-40%) for mastectomy.

*BRIP1* PV carriers have increased OC-risk only, and BC health states were not included. Eligible strategies included RRSO at age 45 years (base case) and no surgery.

### Probabilities

Age-specific OC and BC incidences were derived from published literature for *BRCA1*,^4^ *BRCA2*,^4^ *PALB2*,^5^ *RAD51C*,^6^ and *RAD51D*^6^ PV carriers (eTable 1 in Supplement 1). We used the relative risk for OC^7^ and age-specific OC incidence of the general population^38^ for *BRIP1* PV carriers given insufficiency of *BRIP1*-specific incidence data. For various pathway probabilities and explanations, see eTable 2 and eMethods 1 in Supplement 1.

### Costs

Costs were assessed from payer-perspective (UK National Health Service [NHS]) and reported in 2021 pounds (to convert to US dollars, multiply by 1.38).^30^ The Hospital and Community Health Services Index or NHS cost inflation index were used for inflation adjustment.^39^ The costs of RRSO, RRM, medical prevention, BC surveillance, and cancer treatments (first and subsequent years and terminal care) were derived from National Cost Collection for the NHS^40^ and published literature (eTable 2 and eMethods 2 in Supplement 1).

### Life-Years

A lifetime horizon was adopted to incorporate long-term consequences. All-cause mortality was obtained from UK female life tables from the Office of National Statistics.^41^ Cancer outcomes were modeled using 10-year survival from *BRCA1 *and* BRCA2* PV carriers or women with moderate BC risk for BC^31,42^ and the general population for OC,^32^ with the impact from risk-reducing surgery incorporated. We applied reduced risk of all-cause mortality after RRSO for *BRCA1 *and* BRCA2* PV carriers who did not develop cancer^43,44^ and assumed the same outcomes for other CSG carriers (base case). For detailed survival estimates see eTable 2 and eMethods 3 in Supplement 1.

### Quality-Adjusted Life-Years

Health-state utility scores (per NICE recommendations), which adjusted changes in survival by alterations in quality of life, were used to generate quality-adjusted life-years (QALYs).^30^ Disutilities for RRSO, RRM, and medical prevention were assigned for year of treatment,^45,46^ with disutility of CHD incorporated.^47^ We assigned a disutility for BC surveillance attendance for year of screening and a 1-year disutility for a false-positive result.^45,48^ OC and BC utility scores were derived from published literature^49,50,51,52^ (eTable 2 and eMethods 4 in Supplement 1). All utility scores were age adjusted using the multiplicative method,^53^ combining age-specific utility scores in the healthy state^54^ with utilities in all other health states.

### Statistical Analysis

This study was conducted from December 1, 2022, to August 31, 2023. Costs and health effects were discounted at 3.5%.^30^ The incremental cost-effectiveness ratio (ICER) was calculated as incremental cost per QALY gained and compared with the UK/NICE willingness-to-pay (WTP) threshold of £20 000/QALY to £30 000/QALY.^55^ Net monetary benefit (NMB) was the difference between the monetary value of QALYs (using £20 000/QALY WTP threshold) and costs. OC and BC cases and deaths prevented per 1000 PV carriers were estimated. Several scenario analyses were undertaken, as follows: (1) different ages at surgery; (2) later model starting age (35 years); (3) half HT adherence (40%) following premenopausal RRSO; (4) no change in overall mortality after RRSO for non-*BRCA* CSG carriers; (5) increased OC risk for *BRIP1* (D.G.E., email, April 18, 2023)^7^; and (6) poly (adenosine diphosphate-ribose) polymerase inhibitor (PARP-i) treatment for *BRCA*-altered *ERBB2 *(formerly *HER2*)–negative early BC^56^ and *BRCA*-altered or homologous recombination deficiency (HRD)–positive advanced OC,^57,58^ based on recent guidelines and NICE recommendations.^59,60,61^

Extensive 1-way and probabilistic sensitivity analyses (PSAs) evaluated model uncertainty. Each parameter was varied individually to assess changes in ICERs in 1-way sensitivity analysis. Probabilities and utility scores were varied by 95% CI and range or by ±10%, and costs were varied by ±30%. All parameters were varied simultaneously in the PSA, with assigned distribution (costs: γ distribution; probabilities: β distribution; utility scores: log-normal distribution^62^) over 10 000 simulations. Cost-effectiveness acceptability curves showed the probability that a strategy was cost-effective at varying WTP thresholds. The model was validated using the Assessment of the Validation-Status of Health-Economic decision-models (AdViSHE) tool.^63^ Analyses were conducted using TreeAge Pro.

## Results

### Base Case

The simulated cohort included women aged 30 years with no cancer. RRSO with or without RRM at varied optimal ages was cost-effective for individual *BRCA1*, *BRCA2*, *PALB2*, *RAD51C*,* RAD51D*, and* BRIP1* PV carriers (Table 2). Compared with high-risk BC surveillance and tamoxifen from age 30 years, RRM (at age 30 years), RRSO (at age 35 years), or undergoing both RRM (at 30 years) and RRSO (at 35 years) were cost-effective or cost-saving for *BRCA1* PV carriers. Undergoing both procedures was most cost-effective, with an ICER of −£1942/QALY, providing the largest QALYs (20.84) and NMB (£398 614). For *BRCA2* PV carriers, RRM (at age 35 years), RRSO (at age 40 years), or both RRM (at age 35 years) and RRSO (at age 40 years) were cost-effective or cost-saving compared with high-risk BC surveillance and tamoxifen from age 30 years. RRM plus RRSO yielded the largest QALYs (20.56) and NMB (£394 892) with an ICER of −£89/QALY. RRM (at age 40 years), RRSO (at age 45 years), or both RRM (at age 40 years) and RRSO (at age 45 years) were cost-effective for *PALB2* PV carriers compared with high-risk BC surveillance and tamoxifen from age 30 years. RRM plus RRSO had an ICER of £2381/QALY, with the largest QALYs (20.44) and NMB (£394 369). Compared with moderate-risk BC surveillance and tamoxifen from age 40 years, RRSO at age 45 years was cost-effective for *RAC51C *and *RAD51D* PV carriers with ICERs of £962/QALY and £771/QALY, respectively, yielding larger QALYs (*RAD51C*, 20.49; *RAD51D*, 20.51) and NMB (*RAD51C*, £403 978; *RAD51D*, £404 527). For *BRIP1* PV carriers, RRSO at age 45 years (vs no surgery) yielded 21.03 QALYs, £416 975 NMB, and an ICER of £2355/QALY.

**Table 2.** Lifetime Costs, Health Effects, ICERs, and NMB of Prevention and Surveillance Strategies

Strategy	Costs, £^a^	LYGs	QALYs	NMB, £^a^^,^^b^	ICER, £/QALY^a^
*BRCA1*					
High-risk BC surveillance and tamoxifen from age 30 y^c^	24 767	22.40	17.45	324 295	NA
RRM at age 30 y	25 368	22.67	18.82	350 956	441
RRSO at age 35 y with high-risk BC surveillance and tamoxifen from age 30 y	18 042	24.33	19.11	364 086	−4067
RRM at age 30 y and RRSO at age 35 y	18 190	25.05	20.84	398 614	−1942
*BRCA2*					
High-risk BC surveillance and tamoxifen from age 30 y^c^	16 461	23.43	18.43	352 188	NA
RRM at age 35 y	17 013	23.52	19.42	371 423	558
RRSO at age 40 y with high-risk BC surveillance and tamoxifen from age 30 y	14 214	24.66	19.45	374 842	−2202
RRM at age 35 y and RRSO at age 40 y	16 272	25.00	20.56	394 892	−89
*PALB2*					
High-risk BC surveillance and tamoxifen from age 30 y^c^	10 376	23.64	18.77	365 059	NA
RRSO at age 45 y with high-risk BC surveillance and tamoxifen from age 30 y	11 182	24.75	19.60	380 866	970
RRM at age 40 y	12 260	23.82	19.62	380 160	2219
RRM at age 40 y and RRSO at age 45 y	14 337	24.99	20.44	394 369	2381
*RAD51C*					
Moderate-risk BC surveillance and tamoxifen from age 40 y^c^	4947	23.68	19.59	386 873	NA
RRSO at age 45 y with moderate-risk BC surveillance and tamoxifen from age 40 y	5812	24.92	20.49	403 978	962
*RAD51D*					
Moderate-risk BC surveillance and tamoxifen from age 40 y^c^	4964	23.69	19.61	387 156	NA
RRSO at age 45 y with moderate-risk BC surveillance and tamoxifen from age 40 y	5661	24.94	20.51	404 527	771
*BRIP1*					
No surgery^c^	1520	23.82	20.17	401 958	NA
RRSO at age 45	3525	25.05	21.03	416 975	2355

Abbreviations: BC, breast cancer; ICER, incremental cost-effectiveness ratio; LYGs, life-years gained; NA, not applicable; NMB, net monetary benefit; QALYs, quality-adjusted life years; RRM, risk-reducing mastectomy; RRSO, risk-reducing salpingo-oophorectomy.

^a^ To convert UK pounds to US dollars, multiply by 1.38.

^b^ NMB was calculated using £20 000/QALY willingness-to-pay threshold.

^c^ Reference strategy.

For the population-level outcomes (cancer cases and deaths prevented), see Table 3. Compared with BC surveillance and medical prevention, undergoing both RRM and RRSO (at the same ages as in the base case) could prevent 536 BC cases and 387 OC cases along with 56 BC deaths and 246 OC deaths per 1000 *BRCA1 *PV carriers; 524 BC cases and 162 OC cases along with 67 BC deaths and 103 OC deaths per 1000 *BRCA2 *PV carriers; and 422 BC cases and 42 OC cases along with 102 BC deaths and 28 OC deaths per 1000 *PALB2* PV carriers. RRSO at age 45 years could prevent 102 OC cases and 64 OC deaths per 1000 *RAD51C* PV carriers and 118 OC cases and 76 OC deaths per 1000 *RAD51D* PV carriers relative to BC surveillance and medical prevention. Compared with no surgery, RRSO at age 45 years had 55 fewer OC cases and 37 fewer OC deaths per 1000 *BRIP1* PV carriers.

**Table 3.** Population-Level Outcomes of Prevention and Surveillance Strategies per 1000 Pathogenic Variant Carriers

Strategy	BC cases	BC deaths	OC cases	OC deaths	BC cases prevented	BC deaths prevented	OC cases prevented	OC deaths prevented
*BRCA1*								
High-risk BC surveillance and tamoxifen from age 30 y^a^	601	63	412	253	NA	NA	NA	NA
RRM at age 30 y	83	11	423	260	518	52	−11	−6
RRSO at age 35 y with high-risk BC surveillance and tamoxifen from age 30 y	710	56	24	7	−108	7	388	246
RRM at age 30 y and RRSO at age 35 y	65	7	25	8	536	56	387	246
*BRCA2*								
High-risk BC surveillance and tamoxifen from age 30 y^a^	630	74	171	106	NA	NA	NA	NA
RRM at age 35 y	129	16	173	107	500	57	−2	−1
RRSO at age 40 y with high-risk BC surveillance and tamoxifen from age 30 y	556	34	9	3	74	40	162	103
RRM at age 35 y and RRSO at age 40 y	106	7	9	3	524	67	162	103
*PALB2*								
High-risk BC surveillance and tamoxifen from age 30 y^a^	481	109	46	30	NA	NA	NA	NA
RRSO at age 45 y with high-risk BC surveillance and tamoxifen from age 30 y	402	40	4	2	79	69	42	28
RRM at age 40 y	77	18	47	30	404	91	0	0
RRM at age 40 y and RRSO at age 45 y	59	7	4	1	422	102	42	28
*RAD51C*								
Moderate-risk BC surveillance and tamoxifen from age 40 y^a^	188	53	108	66	NA	NA	NA	NA
RRSO at age 45 y with moderate-risk BC surveillance and tamoxifen from age 40 y	238	48	6	2	−50	6	102	64
*RAD51D*								
Moderate-risk BC surveillance and tamoxifen from age 40 y^a^	174	48	124	78	NA	NA	NA	NA
RRSO at age 45 y with moderate-risk BC surveillance and tamoxifen from age 40 y	220	43	6	2	−46	5	118	76
*BRIP1*								
No surgery^a^	NA	NA	63	40	NA	NA	NA	NA
RRSO at age 45 y	NA	NA	7	3	NA	NA	55	37

Abbreviations: BC, breast cancer; NA, not applicable; OC, ovarian cancer; RRM, risk-reducing mastectomy; RRSO, risk-reducing salpingo-oophorectomy.

^a^ Reference strategy.

### Sensitivity Analyses

**Figure 2.**
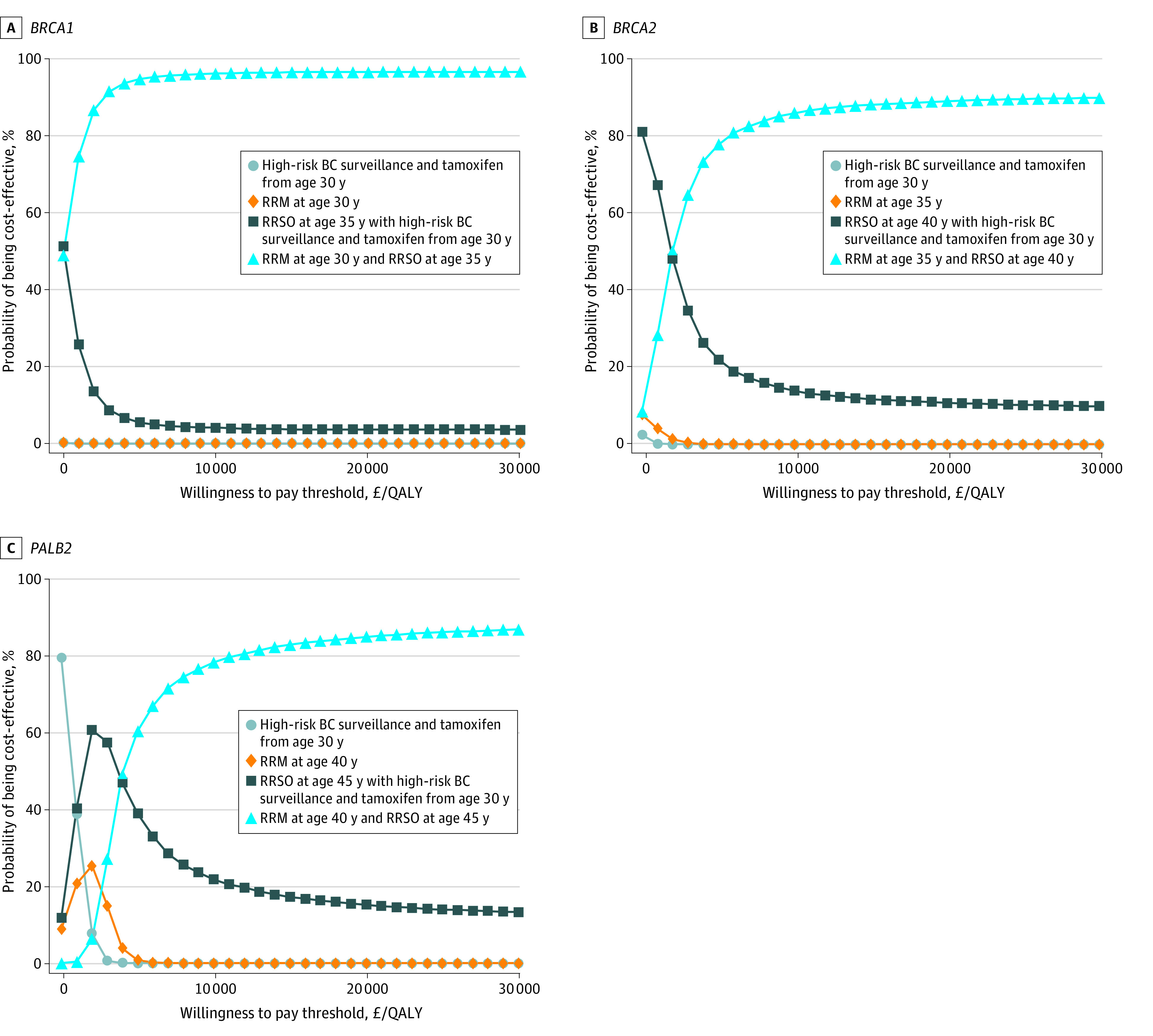
Cost-Effectiveness Acceptability Curves for *BRCA1*, *BRCA2*, and* PALB2* Pathogenic Variant Carriers To convert pounds to US dollars, multiply by 1.38. BC indicates breast cancer; QALY, quality-adjusted life year; RRM, risk-reducing mastectomy; RRSO, risk-reducing salpingo-oophorectomy.

The 1-way sensitivity analyses (eFigure 1 in Supplement 1) showed that model parameters had little influence on base-case results. Despite varying parameters at lower and upper limits, surgical prevention strategies remained cost-effective. The PSA (Figure 2) comparing all strategies simultaneously indicated that at the £20 000/QALY threshold, RRSO plus RRM (at the ages in the base case) was most cost-effective in 96.5% of simulations for *BRCA1 *PV carriers; 89.2% for *BRCA2 *PV carriers; and 84.8% for *PALB2* PV carriers. For *RAD51C*, *RAD51D*, and *BRIP1* PV carriers, RRSO at age 45 years was cost-effective in approximately 100% of simulations (eFigure 2 in Supplement 1).

### Scenario Analyses

The cost-effectiveness of risk-reducing surgery was further supported by various scenario analyses (eTable 3 in Supplement 1). Delaying surgery by 5 years for *BRCA1* (RRM at age 35 years; RRSO at age 40 years) and *BRCA2* (RRM at age 40 years; RRSO at age 45 years) was still cost-effective but with reduced QALYs and NMB and fewer cancers prevented. Conversely, earlier surgery for *BRCA2* (RRM at age 30 years; RRSO at age 35 years) yielded more QALYs (20.94) and a greater NMB (£401 341). Undergoing RRM at age 40 years and RRSO at age 50 years in *PALB2* PV carriers reduced QALYs and cancers prevented, although the NMB was greater. When delayed from age 45 years to 50 years, RRSO yielded fewer QALYs and cancers prevented despite slightly larger NMB for *RAD51C*, *RAD51D*, and *BRIP1* PV carriers. When age of model entry was 35 years, RRSO with or without RRM at respective ages was still cost-effective for each CSG. However, small changes indicated reduced NMB, greater costs, and fewer QALYs for each CSG (eTable 3A in Supplement 1).

Reduced HT adherence (40%) decreased QALYs and NMB for RRSO, although it was still cost-effective compared with nonsurgical interventions. When removing the assumption of changes to overall mortality after RRSO for non-*BRCA* CSG carriers, RRSO was still cost-effective at age 45 years, with reduced QALYs and NMB except for *BRIP1* PV carriers (ICER, £46 103/QALY), but RRSO was cost-effective for *BRIP1* PV carriers (ICER, £15 848/QALY) if undertaken at age 50 years. RRSO at age 45 years for *BRIP1* PV carriers was more cost-effective (ICER, £12 119/QALY) with higher OC risk estimates (relative risk, 5.54).^7^ When incorporating PARP-i treatment for *BRCA-*altered advanced OC, risk-reducing operations were cost-effective (RRSO or RRM plus RRSO were cost-saving) compared with BC surveillance and medical prevention with increased costs and QALYs but reduced NMB. Furthermore, incorporating PARP-i for *BRCA*-altered *ERBB2*-negative early BC, all surgical strategies became cost-saving with reduced NMB. Including PARP-i treatment for *PALB2-*, *RAD51C-*, *RAD51D-*, or* BRIP1*-altered advanced OC^58^ made RRSO at age 45 years cost-saving with reduced NMB (eTable 3 in Supplement 1).

## Discussion

This economic evaluation addressed the important issue of OC and BC risk management. CSG-specific surgical strategies were cost-effective for both *BRCA* and non-*BRCA* CSGs associated with increased OC risk. Undergoing both RRM and RRSO was most cost-effective for *BRCA1* (RRM at age 30 years; RRSO at age 35 years), *BRCA2* (RRM at age 35 years; RRSO at age 40 years), and *PALB2* (RRM at age 40 years; RRSO at age 45 years), potentially preventing 464 to 923 OC and BC cases and 130 to 302 deaths per 1000 PV carriers. RRSO at age 45 years was cost-effective for *RAD51C*, *RAD51D*, and *BRIP1* PV carriers, potentially preventing 55 to 102 OC cases and 37 to 64 OC deaths per 1000 PV carriers. Modeling women entering the model from a later age (35 years) found that risk-reducing surgery remained similarly cost-effective. We found that a one-size-fits-all approach is not appropriate for individual CSG carriers and provided evidence for the most cost-effective risk-reducing operations and their optimal timing, tailored for each CSG. Our results have helped inform the NICE guideline and also support counselling and decision-making for women considering risk-reducing surgery.

RRSO and/or RRM at age 30 to 45 years have been shown to be cost-effective compared with surveillance or no intervention for *BRCA1 *and* BRCA2* carriers previously,^26,27,28^ while disparities in target populations, health care systems, and model assumptions limited generalizability to the UK context. The US National Comprehensive Cancer Network (NCCN)^17^ and the UK Royal College of Obstetricians and Gynaecologists (RCOG)^16^ recommend RRSO at ages 35 to 40 years and 40 to 45 years for *BRCA1* and *BRCA2* PV carriers, respectively. Our base-case analyses (*BRCA1* PV carriers: RRM at age 30 years and RRSO at age 35 years; *BRCA2 *PV carriers: RRM at age 35 years and RRSO at age 40 years) were consistent with these recommendations. When delaying surgery by 5 years, RRSO and/or RRM remained cost-effective but with fewer QALYs and cancers prevented. A further scenario for *BRCA2* PV carriers (RRM at age 30 years; RRSO at age 35 years) yielded more QALYs and a greater NMB with more cancers prevented. This supports possible consideration of and flexibility for earlier age (35-40 years) for RRSO for *BRCA2* PV carriers, especially with early-onset OC in their family history, but decision-making needs to incorporate the impact of menopause at younger than 40 years.

Although RRSO was found cost-effective with a greater than 4% to 5% lifetime OC risk,^64,65^ to our knowledge, no economic evaluations have previously been conducted specifically for *PALB2*, *RAD51C*, *RAD51D*, and *BRIP1* PV carriers. Earlier guidelines have debated the appropriateness and timing of risk-reducing surgery for non-*BRCA* CSG carriers. The American College of Medical Genetics and Genomics (ACMG),^25^ UK Cancer Genetics Group (CGG),^15^ and RCOG^16^ recommend considering RRSO for *PALB2* at ages 50, 50, and 45 to 50 years, respectively, with limited evidence highlighted by NCCN.^17^ RRM should be considered with personalized risk estimates for *PALB2* PV carriers.^15,17,25^ We found RRM (at age 40 years) and/or RRSO (at age 45 years) were cost-effective compared with BC surveillance and medical prevention for *PALB2* PV carriers, and delaying RRSO until age 50 years yielded fewer QALYs and prevented fewer cancers, despite a small increase in NMB. Medical prevention and BC surveillance stops after RRM. The recommended age of RRSO is 40 to 50 years for *RAD51C* and *RAD51D* PV carriers and age 45 to 50 years for *BRIP1* PV carriers, with differences across guidelines.^15,16,17^ Our analysis supports the cost-effectiveness and undertaking of RRSO at age 45 years for *RAD51C*, *RAD51D*, and *BRIP1* PV carriers to maximize QALYs and cancers prevented. Although delaying RRSO to age 50 years (near menopause) slightly increased NMB, this was because additional HT and CHD costs were lower or not needed given postmenopausal status. This needs to be weighed against fewer QALYs and fewer cancers prevented.

Lower HT adherence has been reported after RRSO in some populations and contexts,^66^ which reduces RRSO cost-effectiveness. HT management and compliance is important for symptom control and ameliorating detrimental long-term health consequences, including CHD.^66,67^ Women should be appropriately counselled on the benefits and risks of HT before RRSO. HT adherence appears higher in specialist centers or high-risk familial clinics.^66,68^ Removing the assumed change in overall mortality after RRSO for non-*BRCA* CSG carriers was not associated with significant changes for *PALB2*, *RAD51C*, and *RAD51D* PV carriers, while the ICER of RRSO at age 45 years (but not age 50 years) exceeded the £20 000/QALY to £30 000/QALY threshold for *BRIP1* PV carriers. However, OC risk has been potentially underestimated for *BRIP1* PV carriers due to methodological limitations in published analysis,^7^ and more recent analysis indicates higher lifetime risk (approximately 8% [D.G.E., email, April 18, 2023]). RRSO at age 45 years for *BRIP1* PV carriers becomes more cost-effective with this upper risk estimate,^7^ even without an overall mortality benefit. Better OC risk estimates for *BRIP1* PV carriers and long-term follow-up of non-*BRCA* CSG carriers undergoing RRSO are warranted to clarify these uncertainties.

Recent therapeutic advances offer new treatment options, such as PARP-i therapy. However, these massively increase costs, leading to more complicated trade-offs between prevention and treatment for individuals and the health care system. Given improved overall survival,^56,57^ PARP-i therapy is now recommended by NICE for first-line maintenance of *BRCA*-altered *ERBB2-*negative early BC or advanced OC,^59,60^ and also in OCs with HRD,^61^ given the recently demonstrated overall survival benefit.^58^ We found incorporating PARP-i therapy for advanced OC and *ERBB2*-negative early BC made RRSO and/or RRM cost-saving for *BRCA1 *and *BRCA2* PV carriers. The non-*BRCA* CSGs in our analysis are associated with HRD, and incorporating PARP-i made RRSO more cost-effective (cost-saving in most scenarios) for *PALB2*,* RAD51C*,* RAD51D*, and* BRIP1*. The substantial cost of PARP-i therapy further improves the cost-effectiveness of surgical prevention strategies, emphasizing its importance.

The traditional identification of CSG carriers through cancer genetics clinics is associated with restricted access, and only a small proportion of eligible individuals undergo testing.^69^ Additionally, family history and clinical criteria miss 50% to 80% of CSG carriers. As a result, approximately 97% of PV carriers remain unidentified.^70^ Newer strategies expanding the genetic testing landscape include (1) mainstreaming genetic testing at cancer diagnosis (available for OC^1^ and being piloted for BC^9^) and (2) population genetic testing approaches. Population genetic testing is cost-effective in the Jewish population and the general population for the hereditary breast and ovarian cancer CSGs studied in this article.^71,72,73,74^ Jewish population testing programs were recently implemented in the UK and Israel, and general population studies are ongoing in the UK and Australia.^12,13^ These strategies will detect more PV carriers, and our analysis can facilitate choosing appropriate cancer risk-management strategies tailored to each CSG. Importantly, in our analysis we have used population-based risk estimates, which are corrected for ascertainment.^4^ Using higher risk estimates from familial cancer genetics clinics would make the model more cost-effective.

### Strengths and Limitations

Our study has several strengths. We included non-*BRCA* CSG carriers in cost-effectiveness analysis for the first time of which we are aware and used recent age- and CSG-specific cancer incidence rates.^3,4,5,6,7^ Individual CSG-specific BC surveillance and medical prevention strategies were used as comparators instead of no intervention, which may potentially overestimate the cost-effectiveness of surgical prevention strategies. We adhered to the CHEERS checklist^29^ and NICE recommendations,^30^ and extensive sensitivity and scenario analyses supported the robustness of our results.

Our analysis also has several limitations. We lacked specific cancer incidence data following RRSO and/or RRM for non-*BRCA* CSG carriers and assumed these were similar to *BRCA1 *and* BRCA2* estimates. However, RRSO, even among women with average risk, reduces OC risk by 96% to 97%.^36^ It is likely that OC risk reduction for non-*BRCA* CSG PV carriers and BC risk reduction for *PALB2* PV carriers would be similar to levels obtained for *BRCA*. The estrogen receptor status of *PALB2*-altered BC is similar to *BRCA2*, and most cancers have high-grade histology. Hence, we assumed BC risk reduction following RRSO for *PALB2* was similar to *BRCA2*.^75^ Additionally, our extensive sensitivity analysis showed minimal impact from these assumptions. The ICER using the lower 95% CI value of risk reduction remained well below the NICE WTP threshold of £20 000/QALY (eFigure 1 in Supplement 1). Despite up-to-date data, methodological issues in available studies may lead to OC risk underestimation, especially for *BRCA2* and *BRIP1* PV carriers,^3,4,7^ and more accurate estimates would increase the cost-effectiveness of risk-reducing surgery. Lack of age-specific OC mortality for individual CSG carriers led us to use general population estimates. Although no substantial difference has been reported in the long-term (10-year) survival of *BRCA*-altered vs sporadic OC,^76,77,78^ future studies should incorporate CSG-specific mortality when available. Despite incorporating disutility, all potential harms associated with each intervention may not be fully captured, especially for non-*BRCA* PV carriers. Informed counselling remains critically important. Disutility of risk-reducing surgery was obtained from time–trade-off surveys,^45,46^ and prospective studies measuring disutility using EuroQol (EQ-5D) in women undergoing risk-reducing surgery are needed. Our analysis targeted women in the general population and did not directly evaluate the impact of population diversity and health care disparities due to data insufficiency. Future studies focused on specific population subgroups, especially those underserved, are needed.

The decision about whether and when to undergo risk-reducing surgery is complex and individualized. The uptake and timing of preventive surgery can be varied for eligible PV carriers and increases with time.^22,79^ Sexual dysfunction and menopause symptoms after RRSO and body image and sexual pleasure issues after RRM have been reported, despite reduced cancer distress and unaffected generic quality of life.^67^ Possible surgical complications (RRSO, 3%-5%^80^; RRM, 20% with reconstruction^81,82^) should be factored into counseling to facilitate informed decision-making. Our scenario analyses including age and type of surgery, HT use following premenopausal RRSO, and estimates of cancers prevented further supported and informed counselling of women and decision-making for surgery for each individual carrier. Efforts are needed to ensure uptake in eligible populations along with a focus on ensuring inclusivity and addressing the needs of underserved populations and racial and ethnic minority groups, to realize and maximize the cancer prevention benefits demonstrated in our model. Although risk-reducing early salpingectomy and delayed oophorectomy has the potential to improve sexual function and menopause symptoms (vs premenopausal RRSO),^83^ the level of OC risk reduction, interval cancers, and impact on early menopause remains unknown, and long-term follow-up is needed before considering clinical implementation.^84,85,86^

## Conclusions

Our analysis suggests that undergoing both RRSO and RRM is the most effective and cost-effective option for *BRCA1*, *BRCA2*, and *PALB2* PV carriers, with younger surgery ages for those with higher cancer risk preventing more cancers. RRSO was cost-effective compared with nonsurgical interventions for *RAD51C*, *RAD51D*, and *BRIP1* PV carriers. These findings support personalizing risk-reducing surgery and guideline recommendations and counselling for individual CSG carriers to reduce future OC and BC cases and deaths.

## Notes

Supplement 1. **eTable 1.** Age-Specific Ovarian and Breast Cancer Incidence by Cancer Susceptibility Gene **eTable 2.** Model Parameters **eMethods 1.** Detailed Probabilities Calculation **eMethods 2.** Detailed Cost Calculations **eMethods 3.** Survival of Ovarian or Breast Cancer and Impact of Risk-Reducing Surgery **eMethods 4.** Detailed Utility Score Calculations **eFigure 1.** Tornado Diagrams of 1-Way Sensitivity Analyses **eFigure 2.** Cost-Effectiveness Acceptability Curves for *RAD51C*, *RAD51D*, and *BRIP1* Pathogenic Variant Carriers **eTable 3.** Scenario Analyses Results
eReferences.

Supplement 2.
Data Sharing Statement
