# Supplementary material for: Cost-Effectiveness of Gene-Specific Prevention Strategies for Ovarian and Breast Cancer
Source: JAMA Netw Open. 2024 Feb 9;7(2):e2355324. doi: 10.1001/jamanetworkopen.2023.55324 (PMC10858404; doi:10.1001/jamanetworkopen.2023.55324)
Supplement: Supplement 1. — eTable 1. Age-Specific Ovarian and Breast Cancer Incidence by Cancer Susceptibility Gene eTable 2. Model Parameters eMethods 1. Detailed Probabilities Calculation eMethods 2. Detailed Cost Calculations eMethods 3. Survival of Ovarian or Breast Cancer and Impact of Risk-Reducing Surgery eMethods 4. Detailed Utility Score Calculations eFigure 1. Tornado Diagrams of 1-Way Sensitivity Analyses eFigure 2. Cost-Effectiveness Acceptability Curves for RAD51C, RAD51D, and BRIP1 Pathogenic Variant Carriers eTable 3. Scenario Analyses Results eReferences. [file jamanetwopen-e2355324-s001.pdf]

Supplemental Online Content

Wei X, Sun L, Slade E, et al. Cost-effectiveness of gene-specific prevention strategies for ovarian and breast cancer. *JAMA Netw Open.* 2024;7(2):e2355324. doi:10.1001/jamanetworkopen.2023.55324

eTable 1. Age-Specific Ovarian and Breast Cancer Incidence by Cancer Susceptibility Gene

eTable 2. Model Parameters

eMethods 1. Detailed Probabilities Calculation

eMethods 2. Detailed Cost Calculations

eMethods 3. Survival of Ovarian or Breast Cancer and Impact of Risk-Reducing Surgery

eMethods 4. Detailed Utility Score Calculations

eFigure 1. Tornado Diagrams of 1-Way Sensitivity Analyses

eFigure 2. Cost-Effectiveness Acceptability Curves for *RAD51C*, *RAD51D*, and *BRIP1* Pathogenic Variant Carriers

eTable 3. Scenario Analyses Results

eReferences.

This supplemental material has been provided by the authors to give readers additional information about their work.

eTable 1. Age-Specific Ovarian and Breast Cancer Incidence by Cancer Susceptibility Gene

CSGs	Age	OC incidence per 1,000 person-years (95%CI)	BC incidence per 1,000 person-years (95%CI)	Source
BRCA1	21	–	5.9 (3.4–10.1)	Kuchenbaecker et al[1]
	31	1.8 (1.0–3.4)	23.5 (19.1–28.9)	
	41	7.0 (4.7–10.4)	28.3 (23.1–34.7)	
	51	13.8 (9.2–20.5)	25.7 (19.4–34.0)	
	61	29.4 (19.7–43.8)	25.0 (15.9–39.1)	
	71	5.7 (1.4–22.8)	16.5 (6.2–43.9)	
BRCA2	21	–	4.8 (2.0–11.5)	Kuchenbaecker et al[1]
	31	0.3 (0.1–2.4)	10.8 (7.2–16.2)	
	41	–	27.5 (21.6–35.1)	
	51	6.5 (3.7–11.5)	30.6 (22.8–41.1)	
	61	10.3 (5.5–19.1)	22.9 (13.6–38.7)	
	71	2.3 (0.3–16.3)	21.9 (9.8–48.6)	
PALB2	30	0.09 (0.04–0.2)	2 (1–3)	Yang et al[2]
	40	0.3 (0.1–0.6)	9 (7–11)	
	50	0.7 (0.3–1)	18 (14–22)	
	60	1 (0.6–3)	20 (16–25)	
	70	2 (0.8–4)	19 (14–25)	
	79	2 (1–4)	17 (11–25)	
RAD51C	30	0.05 (0.01–0.2)	0.4 (0.2–0.5)	Yang et al[3]
	40	0.3 (0.2–0.8)	2 (1–3)	
	50	2 (1–3)	5 (3–6)	
	60	7 (4–11)	6 (4–9)	
	70	3 (1–8)	7 (5–10)	
	79	1 (0.2–8)	8 (5–11)	
RAD51D	30	0.03 (0.007–0.1)	0.3 (0.2–0.5)	Yang et al[3]
	40	0.3 (0.1–0.7)	2 (1–2)	
	50	2 (1–3)	4 (3–6)	
	60	6 (4–8)	6 (4–9)	
	70	5 (2–9)	7 (4–10)	
	79	3 (0.9–12)	7 (5–11)	

Note: The OC incidence for *BRIP1* pathogenic variant carriers was calculated by the relative risk of 3.41 (95%CI, 2.12–5.54) for OC[4] and the age-specific OC incidence of the general UK females from Cancer Research UK 2016-2018,[5] due to lack of other data.
Abbreviations: BC, breast cancer; CI, confidence interval; CSGs, cancer susceptibility genes; OC, ovarian cancer.

© 2024 Wei X et al. *JAMA Network Open.*

eTable 2. Model Parameters

Parameters	Base case	95%CI or ranges	Distribution	Source
**Probabilities**				
Risk-reducing interventions and surveillance				
RRM				
BC risk-reduction from RRM without RRSO for *BRCA1/BRCA2*	0.91	0.62–0.98	Beta	Rebbeck et al[6]
BC risk-reduction from RRM without RRSO for *PALB2*	0.91	0.62–0.98	Beta	Assumed (as similar to *BRCA*)
BC risk-reduction from RRM with RRSO for *BRCA1/BRCA2*	0.95	0.78–0.99	Beta	Rebbeck et al[6]
BC risk-reduction from RRM with RRSO for *PALB2*	0.95	0.78–0.99	Beta	Assumed (as similar to *BRCA*)
RRSO				
OC risk-reduction from RRSO for *BRCA1/BRCA2*	0.97	0.80–0.99	Beta	Finch et al[7]; Rebbeck et al[8]; Crosbie et al[9]
OC risk-reduction from RRSO for non-*BRCA* CSGs	0.97	0.86–0.99	Beta	Parker et al[10]
RR for BC from RRSO for *BRCA2**	0.63	0.41–0.97	Beta	Gaba et al[11]
RR for BC from RRSO for *PALB2*	0.63	0.41–0.97	Beta	Assumed (as similar to *BRCA2*)
HRT adherence after premenopausal RRSO	0.80	0.76–0.83	Beta	Read et al[12]
Annual excess risk of CHD after RRSO without HRT	0.0072	0.0068–0.0076	Beta	Parker et al[13]
Annual excess mortality of CHD after RRSO without HRT	0.000879	0.000316–0.001255	Beta	Parker et al[13]
RR for BC mortality from RRSO for *BRCA1*	0.46	0.30–0.70	Beta	Gaba et al[11]
RR for BC mortality from RRSO for *BRCA2*	0.26	0.18–0.39	Beta	Gaba et al[11]
RR for BC mortality from RRSO for *PALB2*	0.26	0.18–0.39	Beta	Assumed (as similar to *BRCA2*)
RR for BC mortality from RRSO for *RAD51C/RAD51D*	0.46	0.30–0.70	Beta	Assumed (as similar to *BRCA1*)
RR for OC mortality from RRSO for *BRCA1/BRCA2*	0.36	0.21–0.60	Beta	Meta-analysis from NICE guideline
RR for OC mortality from RRSO for non-*BRCA* CSGs	0.36	0.21–0.60	Beta	Assumed (as similar to *BRCA*)
RR for overall mortality from RRSO for *BRCA1/BRCA2*	0.36	0.29–0.45	Beta	Meta-analysis from NICE guideline

Parameters	Base case	95%CI or ranges	Distribution	Source
RR for overall mortality from RRSO for non-*BRCA* CSGs	0.36	0.29–0.45	Beta	Assumed in the base-case (as similar to *BRCA*)
Medical prevention				
Uptake of medical prevention	0.163	0.136–0.190	NA	Smith et al[14]
HR for ER-positive BC from tamoxifen	0.66	0.54–0.81	Beta	Cuzick et al[15]
HR for ER-positive BC from anastrozole	0.46	0.33–0.65	Beta	Cuzick et al[16]
BC surveillance				
False positive rate of MRI	0.137	0.083–0.191	NA	Warner et al[17]
False positive rate of mammography	0.053	0.035–0.070	NA	Warner et al[17]
PARP-i (scenario analyses)				
HR for overall mortality of olaparib for *BRCA*-mutated advanced OC	0.55	0.40–0.76	NA	DiSilvestro et al[18]
HR for overall mortality of olaparib plus bevacizumab for HRD-positive (non-*BRCA*) advanced OC	0.71	0.45–1.13	NA	Ray-Coquard 2023[19] (for *PALB2/RAD51C/RAD51D/BRIP1*)
HR for overall mortality of olaparib for *BRCA*-mutated HER2-negative early BC	0.68	0.47–0.97	NA	Geyer et al[20]
Cancer characteristics				
Stage distribution under High-risk BC surveillance				
DCIS	0.2000	NA	NA	Evans et al[21]
Stage 1 BC	0.5436	NA	NA	
Stage 2 BC	0.2462	NA	NA	
Stage 3-4 BC	0.0103	NA	NA	
Stage distribution under Moderate-risk BC surveillance				
DCIS	0.2034	NA	NA	Evans et al[21]
Stage 1 BC	0.4576	NA	NA	
Stage 2 BC	0.3051	NA	NA	
Stage 3-4 BC	0.0339	NA	NA	
Annual recurrence probability of BC				

Parameters	Base case	95%CI or ranges	Distribution	Source
BRCA1	0.0423	NA	NA	Evans et al[21]; Wapnir et al[22]; Anderson et al[23]; Gennari et al[24]; NICE[25,26]
BRCA2	0.0427	NA	NA	
PALB2	0.0427	NA	NA	
RAD51C	0.0421	NA	NA	
RAD51D	0.0427	NA	NA	
Stage 3b-4 OC at diagnosis	0.944	0.727–0.999	Beta	Rosenthal et al[27]
Annual recurrence probability of OC	0.0820	NA	NA	Rosenthal et al[27]; NICE[28]
All-cause mortality	National female life tables	NA	NA	Office for National Statistics[29]
10-year BC survival				
BRCA1	0.912	0.778–0.966	Beta	Evans et al[21]
BRCA2	0.937	0.816–0.979	Beta	
PALB2/RAD51C/RAD51D	0.840	0.810–0.870	Beta	Duffy et al[30]
10-year OC survival				
BRCA1/BRCA2/PALB2/RAD51C/RAD51D/BRIP1	0.353	0.340–0.367	Beta	Cancer research UK[31]
**Costs (2021 GBP)**				
RRM (with reconstruction and complication)	11,768	8,237–15,298	Gamma	NHS reference costs[32]; Neuburger et al[33]; Del Corral et al[34]
RRSO and related costs				
RRSO	4,254	2,978–5,531	Gamma	NHS reference costs[32]
Annual HRT cost	67	47–87	Gamma	Sun et al[35]
DEXA scan	118	83–153	Gamma	NHS reference costs[32]
Osteo-protection	511	358–665	Gamma	Manchanda et al[36]
Annual CHD cost	1,031	722–1,341	Gamma	British Heart Foundation[37]
Fatal CHD	3,312	2,319–4,306	Gamma	NHS reference costs[32]

Parameters	Base case	95%CI or ranges	Distribution	Source
Medical prevention				
Annual tamoxifen cost (premenopausal)	36	25–47	Gamma	BNF[38]
Annual anastrozole cost (postmenopausal)	14	10-18	Gamma	BNF[38]
OC care				
Diagnosis and initial treatment of OC	25,734	18,014–33,455	Gamma	PSSRU[39]; NHS reference costs[32]; NIHR[40]; NICE[28]; BNF[38]
Annual treatment cost of OC 1 to 2 years after diagnosis	18,568	12,998–24,139	Gamma	NHS reference costs[32]; Gilbert et al[41]; Harter et al[42]; Rosenthal[27]; NICE[28]
Annual treatment cost of OC 3 to 10 years after diagnosis	1,560	1,092–2,028	Gamma	
Terminal care of OC	21,070	14,749–27,391	Gamma	Urban et al[43]
BC care				
Screening				
Mammography	60	42–79	Gamma	NICE[44]
MRI	254	178–331	Gamma	NHS reference costs[32]
Diagnosis and initial treatment of BC				
BRCA1	18,378	12,864–23,891	Gamma	NHS reference costs[32]; NICE[26,44]; Sun et al[45]; Jeevan et al[46]; Miller et al[47]; Del Corral et al[34]; Mavaddat et al[48]
BRCA2	18,556	12,989–24,122	Gamma	
PALB2	18,556	12,989–24,122	Gamma	
RAD51C	18,609	13,026–24,192	Gamma	
RAD51D	18,885	13,219–24,550	Gamma	
Annual treatment cost of BC 1 to 10 years after diagnosis				
BRCA1	758	530–985	Gamma	BNF[38]; NICE[26,49-51]; Sun et al[35]; Coleman et al[52]; Mavaddat et al[48]
BRCA2	794	556–1,032	Gamma	
PALB2	849	594–1,104	Gamma	
RAD51C	752	527–978	Gamma	
RAD51D	723	506–940	Gamma	

Parameters	Base case	95%CI or ranges	Distribution	Source
Terminal care of BC	14,277	1,408–43,648	Gamma	Round et al[53]
Yearly cost of olaparib (scenario analyses)	60,462	NA	NA	BNF[38]
Yearly cost of bevacizumab (scenario analyses)	39,845	NA	NA	BNF[38]
**Utility scores**				
Population norms (age-adjusted)	0.692–0.913	0.672–0.923	Log-normal	Szende et al[54]
RRM	0.880	0.760–1.000	Log-normal	Grann et al[55,56]
RRSO	0.950	0.820–1.000	Log-normal	
Medical prevention	0.950	0.810–1.000	Log-normal	
CHD after premenopausal RRSO without HRT	0.670	0.650–0.690	Log-normal	Nyman et al[57]
Mammography surveillance	0.970	0.754–1.000	Log-normal	Grann et al[56]
MRI surveillance	0.960	0.764–1.000	Log-normal	
Disutility from false positive result of BC surveillance	0.105	0.095–0.116	Log-normal	Geuzinge et al[58]; De Haes et al[59]
OC				
Early stage of OC	0.600	0.540–0.660	Log-normal	Havrilesky et al[60]
Advanced stage of OC	0.490	0.441–0.539	Log-normal	
OC recurrence	0.400	0.360–0.440	Log-normal	
OC remission	0.600	0.540–0.660	Log-normal	
Terminal stage of OC	0.160	0.144–0.176	Log-normal	
BC				
DCIS	0.712	0.640–0.783	Log-normal	Robertson et al[61]
Stage 1 BC	0.597	0.537–0.656	Log-normal	
Stage 2 BC	0.527	0.474–0.580	Log-normal	
Stage 3-4 BC	0.497	0.447–0.547	Log-normal	
BC recurrence	0.450	0.215–0.685	Log-normal	Cooper et al[62]

Parameters	Base case	95%CI or ranges	Distribution	Source
BC remission	0.810	0.771–0.849	Log-normal	
Terminal stage of BC	0.160	0.144–0.176	Log-normal	Peasgood et al[63]

Note: *No BC risk-reduction for *BRCA1* following RRSO.
Abbreviations: BC, breast cancer; BNF, British National formulary; CHD, coronary heart disease; CI, confidence interval; CSGs, cancer susceptibility genes; DCIS, ductal carcinoma in situ; DEXA scan, dual energy X-ray absorptiometry scan; ER, oestrogen receptor; HER2, human epidermal growth factor receptor 2; HR, hazard ratio; HRD, homologous recombination deficiency; HRT, hormone replacement therapy; NA, not applicable; NHS, National Health Service; NICE, National Institute for Health and Care Excellence; NIHR, National Institute for Health and Care Research; OC, ovarian cancer; PARP-i, poly (adenosine diphosphate-ribose) polymerase inhibitor; PSSRU, Personal Social Services Research Unit; RR, relative risk; RRM, risk-reducing mastectomy; RRSO, risk-reducing salpingo-oophorectomy.

eMethods 1. Detailed Probabilities Calculation

The breast cancer (BC) risk-reduction from risk-reducing mastectomy (RRM) was taken from the PROSE study for *BRCA1/BRCA2* pathogenic variant (PV)-carriers,[6] which was 91% (95%CI: 62%–98%) and 95% (95%CI: 78%–99%) with or without risk-reducing salpingo-oophorectomy (RRSO), respectively. We assumed the same level of BC risk-reduction with RRM for *PALB2* PV-carriers. The ovarian cancer (OC) risk-reduction from RRSO for *BRCA1/BRCA2* PV-carriers was derived from *BRCA1/BRCA2* related studies[7-9] (97%, 95%CI: 80%–99%), while bilateral salpingo-oophorectomy in average risk general population women in the Nurses' Health Study[10] also reported a similar level of OC risk-reduction (97%, 95%CI: 86%–99%). Hence, we assume the OC risk-reduction for *PALB2/RAD51C/RAD51D/BRIP1* PV-carriers to be similar (97%, 95%CI: 86%–99%) given lack of precise/specific data. There has been a level of uncertainty reported for BC risk-reduction from RRSO,[64-66] and we used the most recent meta-analysis where BC risk-reduction was only observed in *BRCA2* PV-carriers (relative risk: 0.63, 95%CI: 0.41–0.97).[11] Based on a similar proportion of oestrogen-receptor (ER) positive BC for *BRCA2* and *PALB2* PV-carriers,[48] we assumed the same BC risk-reduction from RRSO for *PALB2* PV-carriers. 80% of premenopausal women were assumed to receive hormone replacement therapy (HRT) after RRSO in the base case, given from the surgery age to the average menopause age (51 years).[12] Excess risk of coronary heart disease (CHD) after premenopausal RRSO without HRT was incorporated using data from Nurses' Health Study.[10,13] The excess risk of CHD is calculated by subtracting CHD incidence in women undergoing RRSO from that in women not having this surgery. The excess risk of CHD mortality was also taken from the Nurses' Health Study,[13] where CHD death was reported in 1 out of 33 premenopausal women undergoing RRSO but not taking HRT.

Medical prevention using tamoxifen or anastrozole for 5 years was based on the menopause status.[67] We assume pre-menopausal women use tamoxifen and pos-menopausal women receive anastrozole. The risk-reduction effect was applied to ER-positive BC only, with hazard ratio of 0.66 (95%CI: 0.54–0.81) and 0.46 (95%CI: 0.33–0.65) from the extended long-term follow-up of the IBIS-I BC prevention trial[15] and IBIS-II trial[16], respectively. The compliance of BC surveillance was assumed to be 100%. According to a UK study on enhanced BC surveillance,[21] women with ≥30% lifetime BC-risk undergo mammography and MRI surveillance, and the proportion of women diagnosed as ductal carcinoma in-situ (DCIS), stage 1 BC, stage 2 BC, and stage 3-4 BC was 20.00%, 54.36%, 24.62% and 1.03%, respectively. Women with 17-30% lifetime BC-risk undergo mammography surveillance, with 20.34%, 45.76%, 30.51%, and 3.39% of them diagnosed at DCIS, stage 1 BC, stage 2 BC, and stage 3-4 BC, respectively.[21] False positive rates for MRI and mammography surveillance were included, which were 13.7% and 5.3%, respectively.[17]

eMethods 2. Detailed Cost Calculations

UK costs data were used wherever possible, and the Hospital & Community Health Services Index or NHS cost inflation index were used to convert costs to the year of 2021.[39] Where UK data was unavailable, we used consumer price index (CPI)[68] of original currency and purchasing power parities (PPP)[69] to convert data to 2021GBP.

Cost of risk-reducing strategies

-RRM

The costs of RRM and reconstruction were derived from National Cost Collection for the NHS (financial year 2020/2021, the average unit cost of providing defined services to NHS patients in England).[32] Around 90.6% reconstruction rate has been reported after RRM in the UK population.[33] For RRM and reconstruction, the minor and major complication rate was reported to be 26.2% and 5.6%, respectively.[34] The complication related costs were added to the costs of RRM (minor complication: £475, major complication: £4,331).[34]

-RRSO

The RRSO costs were derived from National Cost Collection for the NHS, using the cost of an upper genital tract laparoscopic or endoscopic intermediate procedure.[32] HRT costs were taken from published UK analyses,[35] which were obtained from the British National Formulary (BNF).[38] Women were assumed to receive one follow-up DEXA scan for monitoring bone health, and calcium and vitamin-D3 for additional osteo-protection.[35] The cost of DEXA scan was taken from National Cost Collection for the NHS,[32] and the osteo-protection cost was taken from Manchanda et al.[36] The HRT, DEXA scan, and osteo-protection costs were only applied to women who underwent RRSO before age 50 years.

CHD costs after premenopausal RRSO was derived from Heart & Circulatory Disease Statistics 2022 from British Heart Foundation.[37] The prevalence of CHD is around 3.2%[37] in the UK with the onset of CHD estimated at age 55 years.[13] The annual cost of CHD was calculated by dividing the cost per capita by the population CHD prevalence. The fatal CHD cost was estimated based on a fatal myocardial infarction from National Cost Collection for the NHS.[32]

-Medical prevention

Tamoxifen 20mg daily or anastrozole 1mg daily was given for 5 years for medical prevention, with uptake rate of 16.3% (obtained from a meta-analysis).[14] The unit cost of tamoxifen and anastrozole was taken from BNF.[38]

Cost of OC

As the treatment for OC is standardised, we applied same OC treatment costs for *BRCA1/BRCA2/PALB2/RAD51C/RAD51D/BRIP1* PV-carriers. The issue of poly (adenosine diphosphate-ribose) polymerase inhibitor (PARP-i) treatment for both *BRCA*-mutated or homologous-recombination-deficiency (HRD) positive advanced OC, is addressed in a scenario analysis. Our approach is conservative in the base-case given the additional significantly high costs of PARP-i.

-Diagnosis costs

OC diagnosis includes pelvic examination, serum CA125 test, transvaginal ultrasound, CT scan, abdominal ultrasound with biopsy, histology and cytology assessment.[28] The cost of pelvic examination was estimated using unit cost from the Personal Social Services Research Unit (PSSRU) and included the cost of GP's and nurse's time.[39] Serum CA125 test cost was

obtained from NICE OC guideline.[28] The transvaginal ultrasound and CT scan costs were taken from National Cost Collection for the NHS,[32] while the costs of abdominal ultrasound-guided biopsy and histology and cytology assessment were obtained from interactive Costing Tool from National Institute for Health and Care Research (NIHR).[40]

-Treatment costs

The OC surgery costs were estimated using reference costs for surgery and chemotherapy. Surgery comprised NHS costs for a complex, open or laparoscopic, upper or lower genital tract procedure for malignancy.[32] Patients were assumed to receive histological assessment of surgical specimen and clinical follow-up after surgery.[32,40] For chemotherapy, patients would receive 6 cycles of carboplatin and paclitaxel. The chemotherapy costs include the appointment for planning chemotherapy, blood test for tumour marker, drug acquisition cost, and administration cost, derived from the National Cost Collection for the NHS,[32] the NIHR interactive Costing Tool,[40] the NICE OC guideline,[28] and BNF.[38] During the treatment, women were assumed to receive two CT scans to monitor disease progression. Scenario analysis of using PARP-i (olaparib) for *BRCA*-mutated advanced OC, or olaparib plus bevacizumab for HRD-positive (non *BRCA*-mutated) advanced OC was conducted according to NICE recommendation.[70,71] The list price of olaparib was £2,317.5/14-day pack from BNF,[38] with average treatment duration of 2 years.[18,19] Bevacizumab was given for an average duration of 15 months,[19] with the list price of £205.55 for 100mg/4ml.[38]

-Recurrence costs

The proportion of OC diagnosed at stage 3b-4 was 94.4% for women with increased OC-risk who did not receive surveillance, according to the UK Familial Ovarian Cancer Screening Study (Phase II).[27] The recurrence rate of early and advanced OC was assumed to be 10%

and 90%, respectively.[28] The chemotherapy costs for OC recurrence were derived from Gilbert et al,[41] and 25% women with recurrence were assumed to receive secondary cytoreduction surgery based on the DESKTOP II trial.[42] 80% recurrence was assumed to occur in the first two years after active treatment.[28]

-Follow up and terminal care costs

Women were assumed to receive four consultant visits, four CA125 tests, and one CT scan each year for the first two years after active treatment.[28] From the third year, women would receive two consultant visits, two CA125 tests, and one CT scan each year.[28] The OC terminal care costs for the last 90 days prior to death was derived from Urban et al.[43]

Cost of BC

BC costs for different CSGs were adjusted for the differences in stage distribution, the proportion of being ER-positive, human epidermal growth factor receptor 2 (HER2)-positive, or lymph node positive. Based on the BRIDGES study,[48] 31%, 75%, 73%, 50%, 65% BC were ER-positive, while 10%, 14%, 22%, 9%, 4% BC were HER2-positive for *BRCA1*, *BRCA2*, *PALB2*, *RAD51C*, *RAD51D* PV-carriers, respectively. The proportion of lymph node positive BC was 36%, 47%, 47%, 33%, 50% for *BRCA1*, *BRCA2*, *PALB2*, *RAD51C*, *RAD51D* PV-carriers, respectively.[48] 49% BC were assumed to be premenopausal.[35]

-Screening and diagnosis costs

BRCA1/BRCA2/PALB2 PV-carriers were assumed to receive annual MRI from age 30–49 years and annual mammography from age 40–69 years (High-risk BC surveillance), while *RAD51C/RAD51D* PV-carriers were assumed to receive annual mammography from age 40–59 years, and then routine triennial mammography as per NHS BC screening programme

from age 60 years (Moderate-risk BC surveillance).[67] BC diagnosis was made by the combination of clinical examination, mammography, and biopsy. The costs of clinical breast examination, mammography, MRI scan, ultrasound guided core needle biopsy were derived from National Cost Collection for the NHS[32] and NICE familial BC costing report[44].

-Pre-treatment axilla ultrasound costs

Pretreatment ultrasound evaluation of the axilla should be offered to all patients being investigated for early invasive cancer.[51] Ultrasound-guided needle biopsy should be offered if morphologically abnormal lymph nodes are identified (around 33% of women with early invasive BC).[26,51] The commissioning cost of pre-treatment ultrasound evaluation of the breast and axilla was the same as that of the breast only.[26] Therefore, only the cost of ultrasound-guided needle sampling was included for the costing model, taken from National Cost Collection for the NHS.[32]

-Sentinel lymph node biopsy (SLNB) costs

SLNB is the preferred technique for staging the axilla for early invasive BC and no evidence of lymph node involvement on ultrasound or a negative ultrasound-guided needle biopsy.[51] 87% , 68%, and 38% of stage 1, stage 2, and stage 3-4 BC were assumed to undergo this procedure, based on a BC costing study in England.[45] The SLNB cost was obtained from National Cost Collection for the NHS,[32] including the sentinel lymph node scan and unilateral intermediate breast procedure.

-Axillary lymph node dissection (ALND) costs

ALND should be offered to women with positive axillary lymph node BC.[51] Cost of ALND is assumed to be 25% of the cost of breast surgery according to NICE BC guideline development group recommendation.[25]

-Breast surgery costs

Breast surgery include breast conserving surgery and mastectomy. All women with non-invasive BC were assumed to undergo breast conserving surgery. 78%, 52%, and 33% of stage 1, stage 2, and stage 3-4 BC would undergo breast conserving surgery, while 16%, 35%, and 67% would undergo mastectomy (with/without reconstruction).[45] 23.3% reconstruction rate following mastectomy was reported for UK BC patients.[46] The minor and major complication rate was reported to be 19.5% and 2.0% following mastectomy alone, and 24.5% and 4.1% following mastectomy and reconstruction.[47] The breast surgery costs were derived from National Cost Collection for the NHS,[32] and the minor or major complications costs from published literature.[34]

-Chemotherapy and radiotherapy costs

Adjuvant therapy is offered to women with invasive BC who are not at low-risk as per NICE BC guidelines.[49,51] Chemotherapy was based on polychemotherapy, including costs of planning, administration, first-line, second-line, and third-line (where applicable) therapy and related toxicity management, derived from NICE advanced BC guideline.[49] 9%, 24%, and 44% of stage 1, stage 2, and stage 3-4 BC were assumed to receive chemotherapy.[45] Radiotherapy is offered at a treatment centre 5 days a week for 3 weeks, using external beam radiotherapy giving 40 Gy in 15 fractions.[51] 70%, 61%, and 83% of stage 1, stage 2, and stage 3-4 BC would receive radiotherapy.[45] Costs of planning and radiotherapy were taken from National Cost Collection for the NHS.[32]

-Endocrine therapy costs

Women with ER-positive BC would receive endocrine therapy, with tamoxifen 20mg daily (premenopausal) or anastrozole 1mg daily (postmenopausal) according to NICE BC guidelines.[49,51] The duration of endocrine therapy was assumed to be 5 years. The unit cost of tamoxifen and anastrozole was obtained from BNF,[38] and the ER test cost (for all invasive cancers) was taken from a local NHS trust.[35]

-Biological therapy costs

Women with HER2-positive invasive BC are eligible for adjuvant trastuzumab therapy, given at 3-week intervals for 1 year or until recurrence.[51] 10% of the eligible patients were assumed to be intolerant of trastuzumab because of the risk of adverse events.[26] For patients suitable for trastuzumab treatment, 80% would receive the therapy.[26] It was assumed that 80% patients taking trastuzumab would experience disease progression outside the central nervous system, and 50% would continue taking trastuzumab.[26] The HER-2 test cost (for all invasive cancers) was taken from a local NHS trust.[35] The trastuzumab costs including administration and cardiac monitoring were £19,770 per patient, taken from NICE BC costing report.[26]

-PARP-i costs

Olaparib is recently recommended as an option for the adjuvant treatment of *BRCA*-mutated HER2-negative high-risk early BC that has been treated with neoadjuvant or adjuvant chemotherapy by NICE.[72] Olaparib treatment was only included in a scenario analysis for *BRCA1/BRCA2* PV-carriers. The list price of olaparib was taken from BNF,[38] and the duration of treatment was assumed to be 1 year.[20]

-Bisphosphonate costs

Bisphosphonates should be considered for patients newly diagnosed with bone metastases to prevent skeletal-related events and reduce pain.[49] 74% advanced BC patients were assumed to develop bone metastases, and 87% of those with bone metastases were offered bisphosphonates.[26,50,73] The commonly used bisphosphonates included oral sodium clodronate, oral ibandronic acid, intravenous zoledronic acid, and intravenous pamidronate disodium,[26,51] and the proportion of patients receiving these drugs was 20%, 30%, 25% and 25%, respectively.[26] The annual drug costs (including administration costs) were £2,348, £3,333, £4206, and £3,734, respectively, taken from NICE BC costing report.[26] The bisphosphonate treatment duration was assumed to be 2 years, which was based on the life expectancy of advanced BC with bone metastases.[52]

-Recurrence costs

For non-invasive BC, there was a 25% risk of local recurrence over 10 years and half of these recurrences would be invasive cancer.[25] For early and locally advanced BC, the locoregional recurrence rate was 15.9% for node-positive disease,[22] and 11.0% for node-negative disease.[23] Based on the proportion of lymph node positive BC,[48] the weighted locoregional recurrence rate was 12.76%, 13.30%, 13.30%, 12.62%, and 13.45% for *BRCA1, BRCA2, PALB2, RAD51C,* and *RAD51D* PV-carriers, respectively. The distant recurrence rate was 35% for early and locally advanced BC.[26] The recurrence rate for advanced BC was reported to be 66.3%, derived from the 33.7% relapse-free 5-year survival.[24]

-Follow-up and terminal care costs

After active BC treatment, patients were assumed to receive clinical follow-up every four months in the first two years, every six months from the third to the fifth year, and every year

from the sixth to the tenth year. They were also offered annual mammography surveillance for ten years. The cost of clinical follow-up was obtained from National Cost Collection for the NHS.[32] The terminal care costs for BC in the last year prior to death were derived from a UK study by Round et al.[53]

eMethods 3. Survival of Ovarian or Breast Cancer and Impact of Risk-Reducing Surgery

As no statistically significant difference was reported in the long-term survival between CSG-associated and sporadic OC,[74-77] we used the 10-year OC survival of 35.3% (95%CI: 34.0%–36.7%) in general population from Cancer Research UK.[31] BC survival under mammography and MRI surveillance was taken from a UK study for 14,311 women by Evans et al,[21] and the 10-year survival was 91.2% (95%CI: 77.8%–96.6%) for *BRCA1* and 93.7% (95%CI: 81.6%–97.9%) for *BRCA2* PV-carriers, respectively. The BC survival for *PALB2/RAD51C/RAD51D* PV-carriers was derived from the FH01 study on mammographic surveillance for moderate BC-risk UK women, where 10-year survival of 84.0% (95%CI: 81.0%–87.0%) was reported.[30] Women were considered long-term survivors if they were alive without evidence of recurrence 10 years after cancer diagnosis. Long-term cancer survivors were assumed to have the same probability of death as the general population.

The impact of risk-reducing surgery on cancer survival was included in our model. The BC survival after RRSO for *BRCA1/BRCA2* PV-carriers was taken from a recent meta-analysis.[11] Based on BC pathological features,[48] we applied the same impact on BC survival from RRSO for *PALB2* PV-carriers as that for *BRCA2* PV-carriers, and for *RAD51C/RAD51D* PV-carriers as that for *BRCA1* PV-carriers. The OC survival after RRSO for *BRCA1/BRCA2* PV-carriers was obtained from a meta-analysis conducted for the NICE guideline, which was also assumed for *PALB2/RAD51C/RAD51D/BRIP1* PV-carriers. A reduction in overall mortality after RRSO was reported for *BRCA1/BRCA2* PV-carreirs.[78,79] Hence, we applied reduced overall mortality after RRSO for *BRCA1/BRCA2* PV-carriers who did not develop cancer, and we also assumed the same impact for other CSG-carriers associated with an increased risk of OC in the base-case as CSG-specific data are lacking. This issue was further explored

in a scenario analysis with no impact on overall mortality in other (non-*BRCA*) OC CSG-carriers. Mixed evidence exists on overall mortality after RRSO in general population women, with decreased OC specific mortality but potentially increased all-cause mortality reported mainly in pre-menopausal women who do not take HRT after oophorectomy.[13,80-83] Additionally no added risk of overall mortality has also been reported.[84]

For the scenario analysis of using PARP-i (olaparib), the hazard ratio for overall survival was 0.68 (98.5% CI: 0.47–0.97) for *BRCA*-mutated HER2-negative early BC, taken from the pre-specified second interim analysis of the OlympiA phase III trial.[20] Improvement in overall survival after olaparib for *BRCA*-mutated advanced OC (hazard ratio: 0.55, 95% CI: 0.40–0.76) was taken from the SOLO1/GOG 3004 Trial.[18] Overall survival benefit was recently reported for primary maintenance treatment with olaparib plus bevacizumab for HRD-positive advanced OC (hazard ratio: 0.62, 95%CI: 0.45–0.85) from the prespecified final overall survival analysis for the PAOLA-1/ENGOT-ov25 trial.[19] For our analysis we used the overall survival of olaparib plus bevacizumab treatment for HRD-positive "not *BRCA*-mutated" advanced OC (hazard ratio: 0.71, 95%CI: 0.45–1.13) for *PALB2/RAD51C/RAD51D/PALB2* PV-carriers.[19]

eMethods 4. Detailed Utility Score Calculations

Quality-adjusted life-years (QALYs) are the recommended generic measure of health benefit by NICE, which reflects both mortality and health-related quality-of-life effects.[85] It equals time spent in a specific health state multiplied by the corresponding utility score. Utility score is an indicator of individual preference for a specific health state, where '1' implies perfect health and '0' implies death. Utility score is used for quality-of-life adjustment for different health states included in the model.

Utility scores of prevention and surveillance strategies were derived from time trade-off (TTO) surveys among women with or without *BRCA1/BRCA2* PV.[55,56] Utility scores of RRM, RRSO, medical prevention, mammography surveillance, and MRI surveillance were 0.88, 0.95, and 0.95, 0.97, and 0.96 for *BRCA1/BRCA2* PV-carriers, respectively. We also applied these utility scores to non-*BRCA* PV-carriers due to lack of data. The disutility of RRSO and RRM were applied during the year of surgery, and disutility of CHD after premenopausal RRSO was included, which was taken from an EQ-5D-3L survey by Nyman et al.[57] The disutility of BC surveillance attendance was applied for the year of screening, and a disutility of 0.105 from a visual analogue scale (VAS) survey was further assigned in case of a false positive result for one year.[58,59]

Utility scores of OC were derived from a survey of OC patients and females of general public.[60] Utilities were reported by both TTO and VAS in this study, and we chose the estimates using TTO as visual scales comparing health state preferences were subject to inherent biases and usually less accurate.[86] Utility scores of early, advanced, recurrent, remittent, and terminal OC were 0.600, 0.490, 0.400, 0.600, and 0.160, respectively.[60] BC utility scores were derived from a NIHR health technology assessment conducted by

Robertson et al,[61] where utility scores of different BC stages were taken from a systematic review and adjusted for the decrement from chemotherapy. Utility scores of DCIS, stage 1, stage 2, and stage 3-4 BC were 0.712, 0.597, 0.527, and 0.497, respectively.[61] Utility scores of recurrent and remittent BC were derived from the pooling of utilities from studies using health state descriptions or standard gamble by Cooper et al,[62] which was 0.450 and 0.810, respectively. Utility score of terminal BC (0.160) was derived from a literature review on utility scores of BC related health states.[63]

All health state utility scores were age-adjusted using multiplicative method,[87] which combined age-specific utility scores in the 'healthy' state with utilities in all other health states. The population norms of utility scores were taken from the Health Survey for England, where EQ-5D-3L questionnaire was used.[54]

eFigure 1. Tornado Diagrams of 1-Way Sensitivity Analyses

eFigure-1a. Tornado diagrams for *BRCA1* pathogenic variant carriers

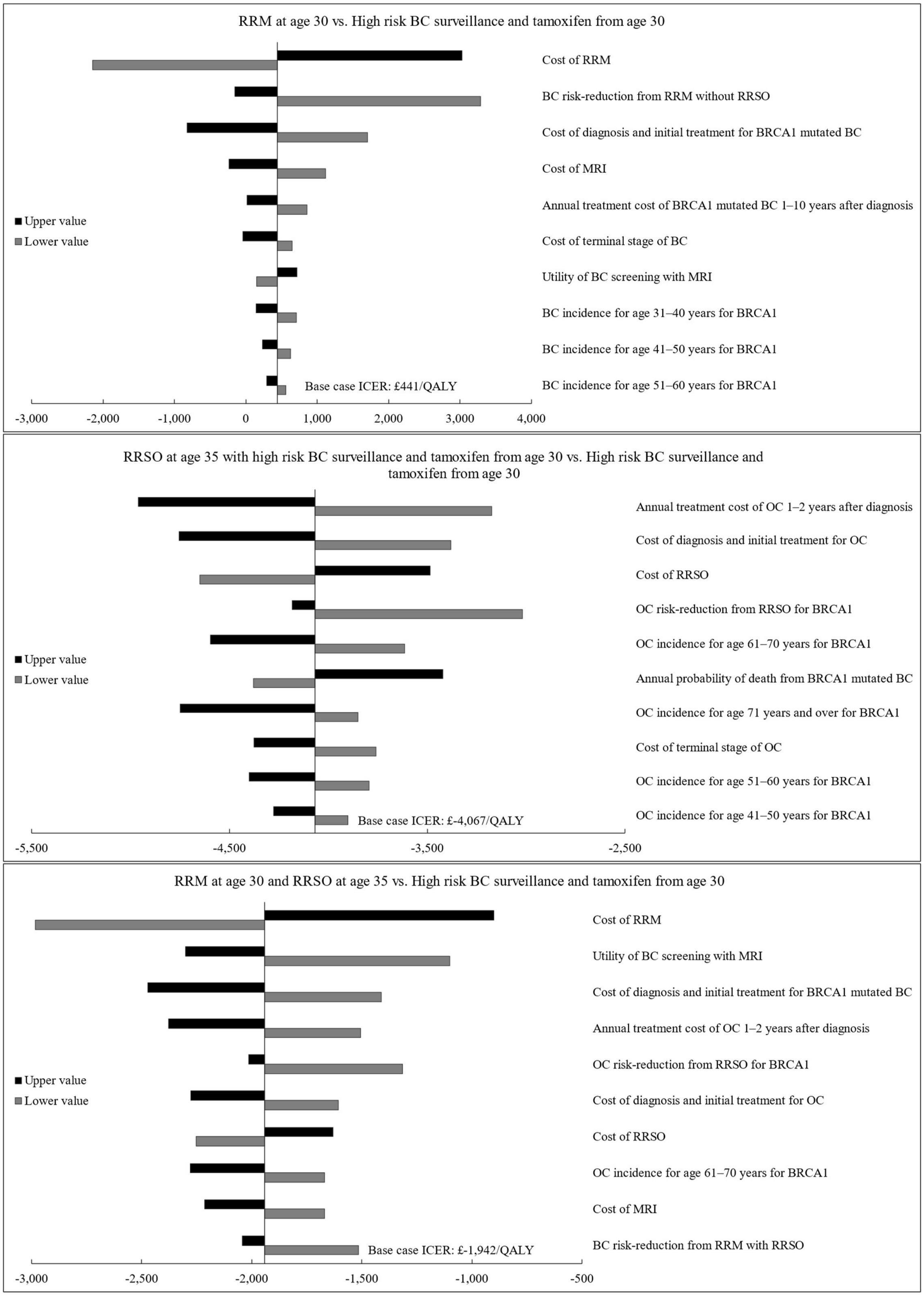

eFigure-1b. Tornado diagrams for *BRCA2* pathogenic variant carriers

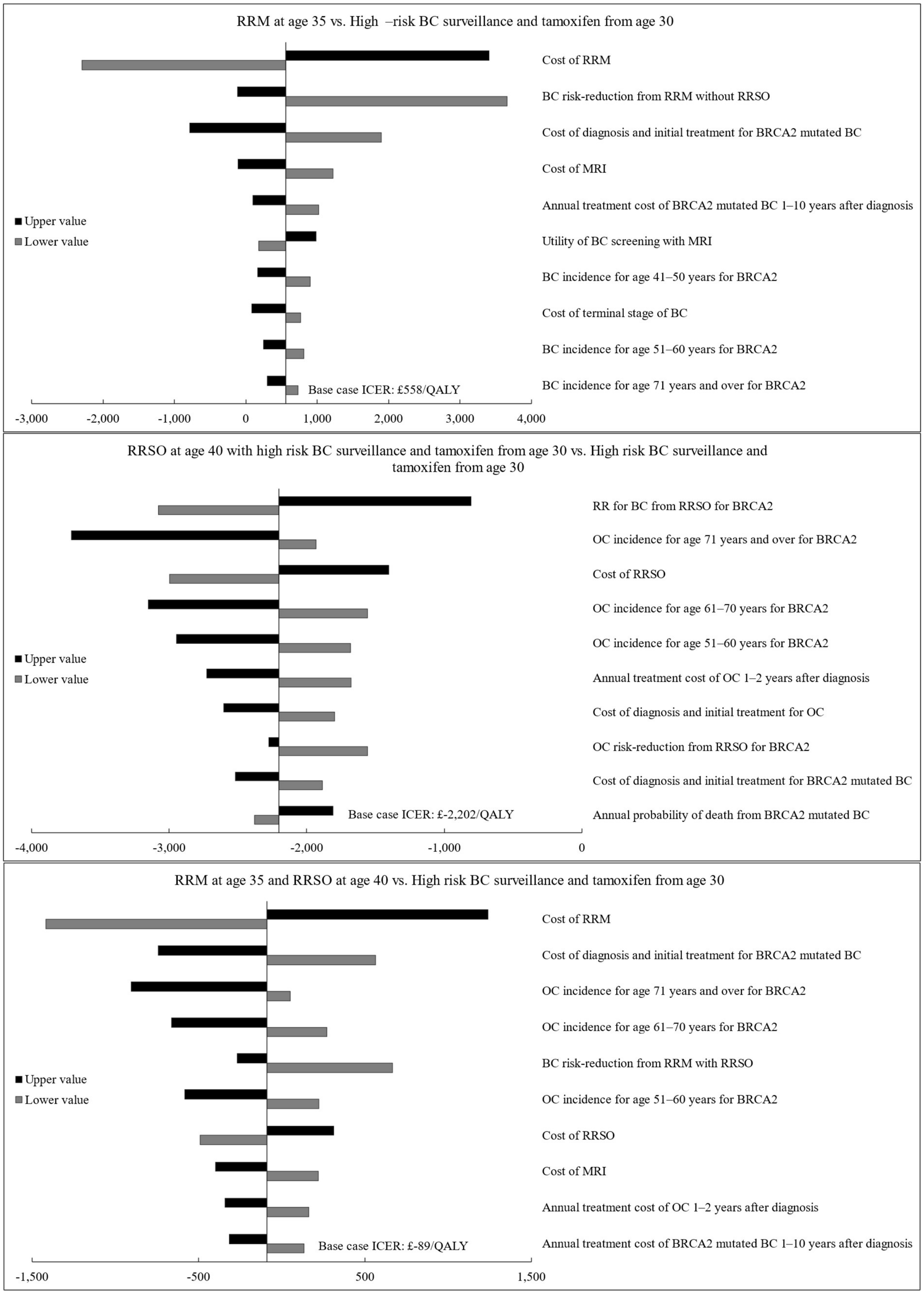

eFigure-1c. Tornado diagrams for *PALB2* pathogenic variant carriers

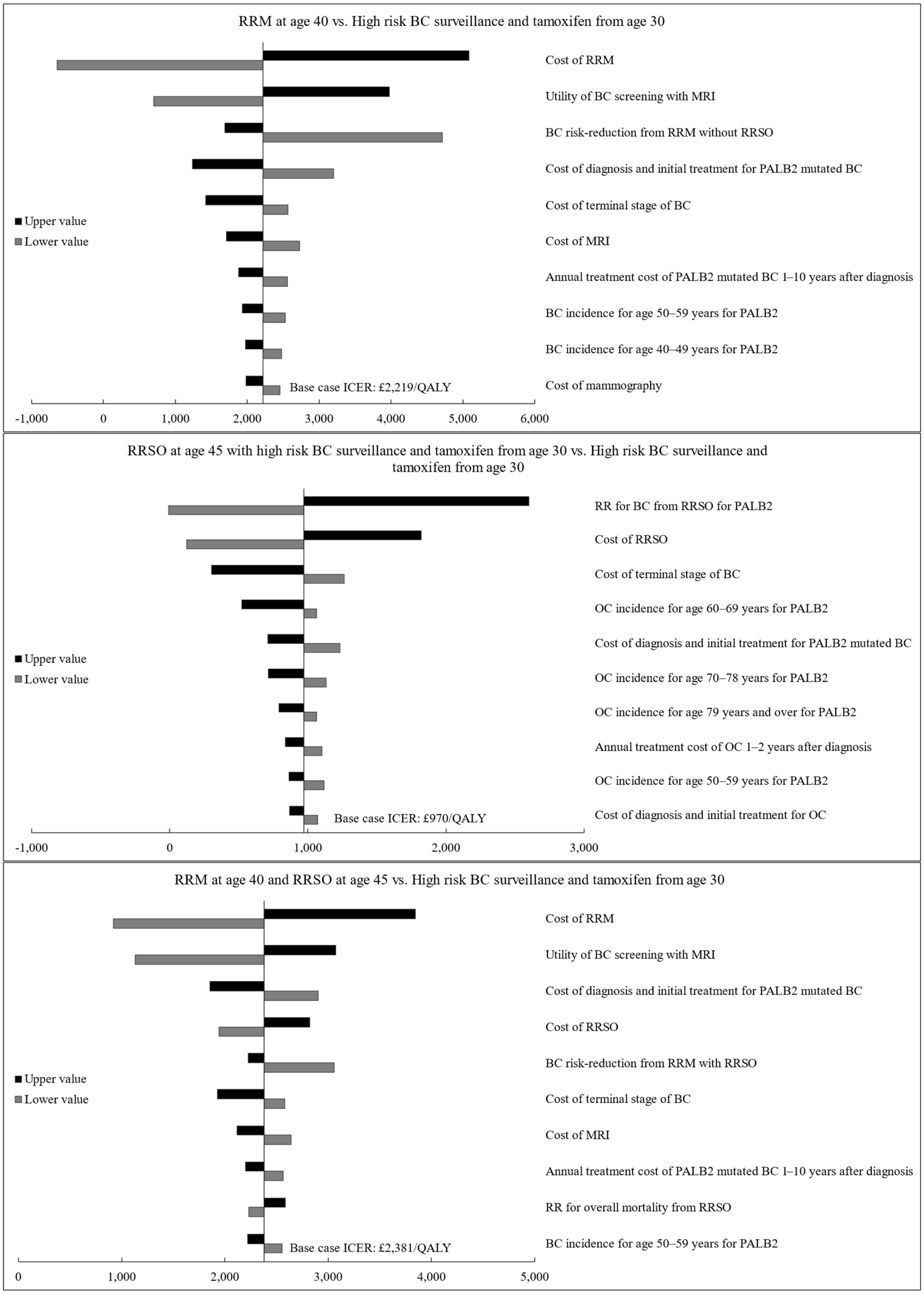

eFigure-1d. Tornado diagram for *RAD51C* pathogenic variant carriers

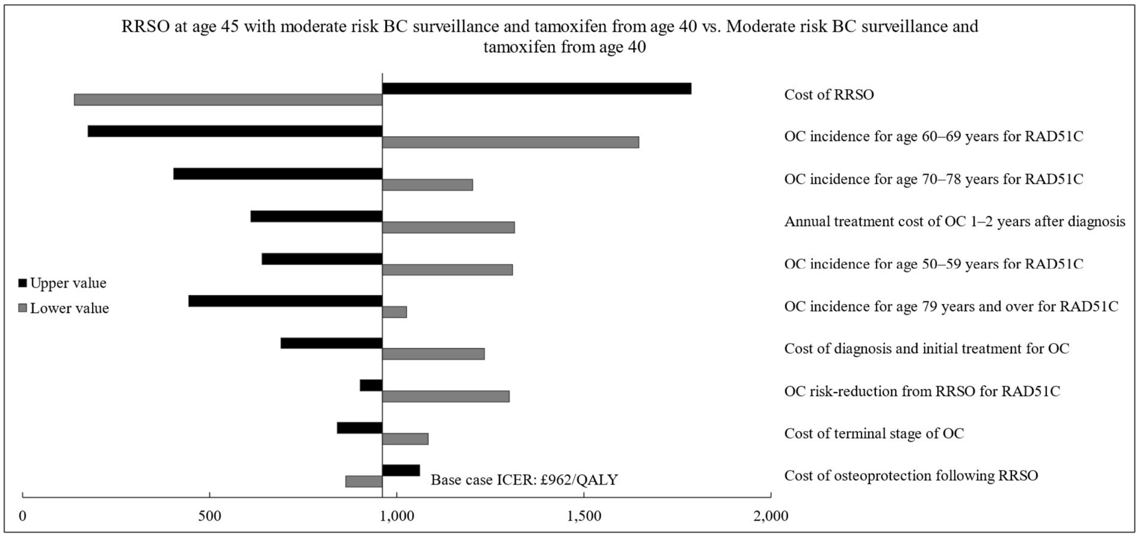

eFigure-1e. Tornado diagram for *RAD51D* pathogenic variant carriers

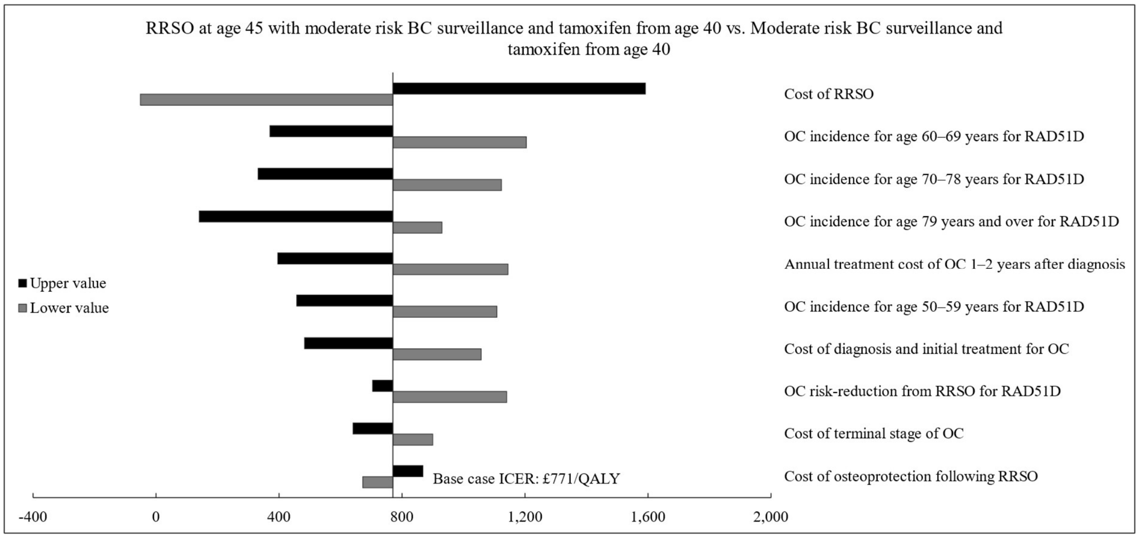

eFigure-1f. Tornado diagram for *BRIP1* pathogenic variant carriers

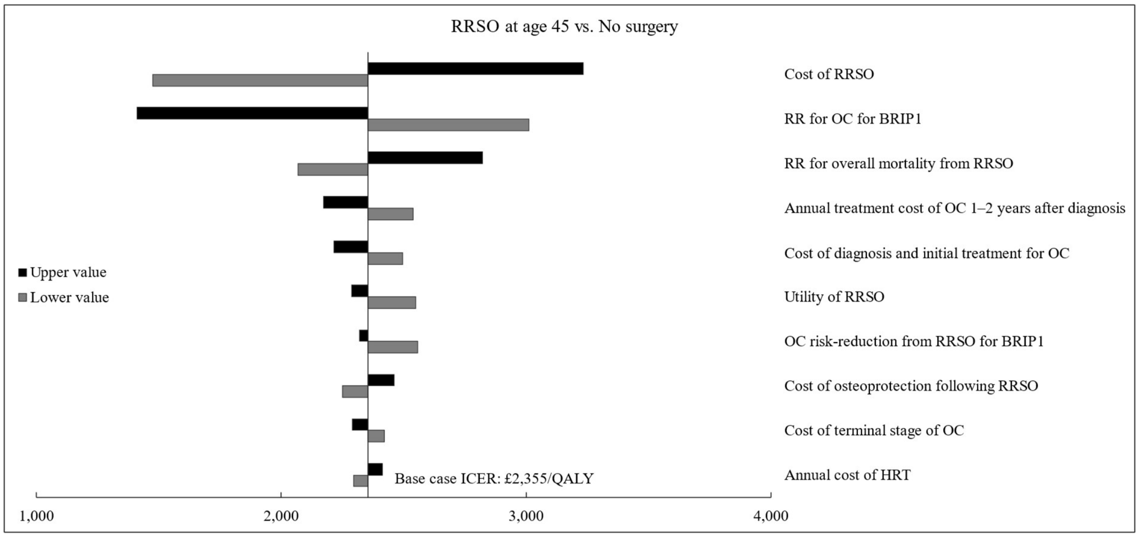

Abbreviations: BC, breast cancer; HRT, hormone replacement therapy; ICER, incremental cost-effectiveness ratio; OC, ovarian cancer; QALY, quality-adjusted life-year; RR, relative risk; RRM, risk-reducing mastectomy; RRSO, risk-reducing salpingo-oophorectomy.

eFigure 2. Cost-Effectiveness Acceptability Curves for *RAD51C*, *RAD51D*, and *BRIP1* Pathogenic Variant Carriers

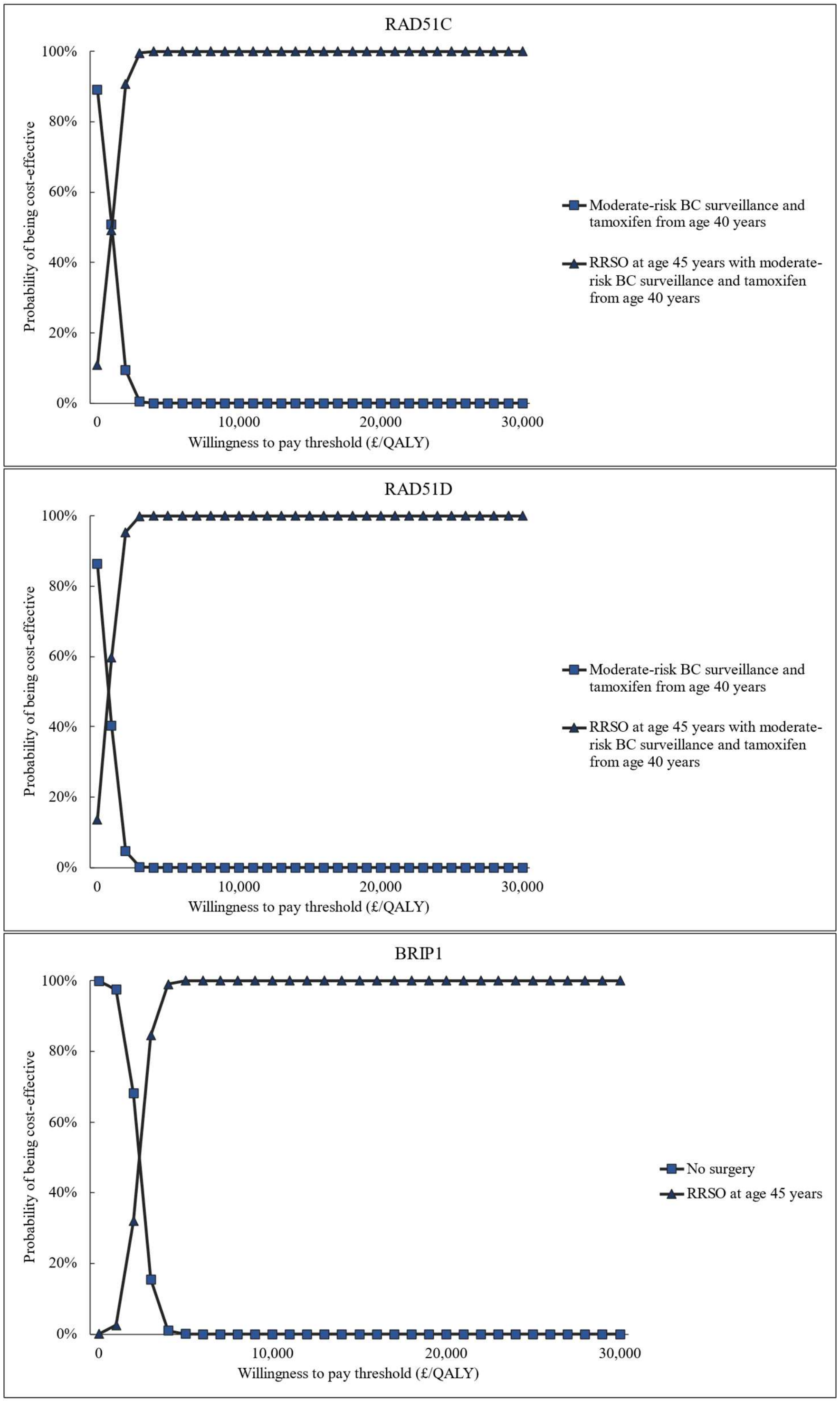

Abbreviations: BC, breast cancer; QALY, quality-adjusted life year; RRSO, risk-reducing salpingo-oophorectomy.

eTable 3. Scenario Analyses Results

eTable-3a. Lifetime costs, health effects, ICER and NMB of prevention and surveillance strategies for scenario analyses

Strategy	Costs (£)	LYGs	QALYs	NMB[a] (£)	ICER (£/QALY)
**Ages of surgery**					
***BRCA1*: older ages of surgery**					
High-risk BC surveillance and tamoxifen from age 30[b]	24,767	22.40	17.45	324,295	
RRM at age 35	25,377	22.59	18.42	343,121	628
RRSO at age 40 with high-risk BC surveillance and tamoxifen from age 30	17,404	24.16	18.96	361,824	-4,882
RRM at age 35 and RRSO at age 40	17,620	24.74	20.26	387,598	-2,545
***BRCA2*: younger ages of surgery**					
High-risk BC surveillance and tamoxifen from age 30[b]	16,461	23.43	18.43	352,188	
RRSO at age 35 with high-risk BC surveillance and tamoxifen from age 30	14,914	24.76	19.54	375,840	-1,400
RRM at age 30	17,264	23.55	19.72	377,188	623
RRM at age 30 and RRSO at age 35	17,428	25.14	20.94	401,341	386
***BRCA2*: older ages of surgery**					
High-risk BC surveillance and tamoxifen from age 30[b]	16,461	23.43	18.43	352,188	
RRM at age 40	16,875	23.49	19.17	366,620	557
RRSO at age 45 with high-risk BC surveillance and tamoxifen from age 30	13,686	24.54	19.35	373,362	-3,016
RRM at age 40 and RRSO at age 45	15,413	24.84	20.22	388,963	-587
***PALB2*: older ages of surgery**					
High-risk BC surveillance and tamoxifen from age 30[b]	10,376	23.64	18.77	365,059	
RRSO at age 50 with high-risk BC surveillance and tamoxifen from age 30	10,431	24.65	19.55	380,471	71
RRM at age 40	12,260	23.82	19.62	380,160	2,219
RRM at age 40 and RRSO at age 50	13,315	24.94	20.43	395,231	1,775
***RAD51C*: older ages of surgery**					

Strategy	Costs (£)	LYGs	QALYs	NMB[a] (£)	ICER (£/QALY)
Moderate-risk BC surveillance and tamoxifen from age 40[b]	4,947	23.68	19.59	386,873	
RRSO at age 50 with moderate-risk BC surveillance and tamoxifen from age 40	4,753	24.87	20.48	404,833	-219
***RAD51D*: older ages of surgery**					
Moderate-risk BC surveillance and tamoxifen from age 40[b]	4,964	23.69	19.61	387,156	
RRSO at age 50 with moderate-risk BC surveillance and tamoxifen from age 40	4,609	24.88	20.50	405,361	-399
***BRIP1*: older ages of surgery**					
No surgery[b]	1,520	23.82	20.17	401,958	
RRSO at age 50	2,530	25.00	21.01	417,762	1,201
**Model starting age of 35 years**					
***BRCA1*: entering model at age 35**					
High-risk BC surveillance and tamoxifen from age 35[b]	26,311	21.39	16.29	299,515	
RRM at age 35	27,103	21.63	17.57	324,292	620
RRSO at age 35 with high-risk BC surveillance and anastrozole from age 35	18,716	23.70	18.30	347,196	-3,789
RRM at age 35 and RRSO at age 36	18,463	24.43	19.95	380,455	-2,147
***BRCA2*: entering model at age 35**					
High-risk BC surveillance and tamoxifen from age 35[b]	17,405	22.48	17.32	328,988	
RRM at age 35	18,071	22.59	18.55	352,931	542
RRSO at age 40 with high-risk BC surveillance and tamoxifen from age 35	14,831	23.99	18.56	356,461	-2,067
RRM at age 35 and RRSO at age 40	17,385	24.41	19.94	381,445	-8
***PALB2*: entering model at age 35**					
High-risk BC surveillance and tamoxifen from age 35[b]	10,713	22.71	17.69	343,023	
RRSO at age 45 with high-risk BC surveillance and tamoxifen from age 35	11,691	24.05	18.68	362,008	980
RRM at age 40	12,977	22.92	18.70	361,063	2,231
RRM at age 40 and RRSO at age 45	15,484	24.34	19.68	378,133	2,393
***RAD51C*: entering model at age 35**					

**Strategy**	**Costs (£)**	**LYGs**	**QALYs**	**NMB[a] (£)**	**ICER (£/QALY)**
Moderate-risk BC surveillance and tamoxifen from age 40[b]	5,818	22.74	18.35	361,203	
RRSO at age 45 with moderate-risk BC surveillance and tamoxifen from age 40	6,855	24.21	19.42	381,598	968
***RAD51D*: entering model at age 35**					
Moderate-risk BC surveillance and tamoxifen from age 40[b]	5,859	22.75	18.37	361,459	
RRSO at age 45 with moderate-risk BC surveillance and tamoxifen from age 40	6,693	24.23	19.44	382,161	775
***BRIP1*: entering model at age 35**					
No surgery[b]	1,712	22.91	19.05	379,290	
RRSO at age 45	4,101	24.38	20.06	397,188	2,355
**HRT adherence following premenopausal RRSO**					
***BRCA1*: 40% HRT adherence**					
High-risk BC surveillance and tamoxifen from age 30[b]	24,767	22.40	17.45	324,295	
RRM at age 30	25,368	22.67	18.82	350,956	441
RRSO at age 35 with high-risk BC surveillance and tamoxifen from age 30	17,913	24.31	19.07	363,397	-4,251
RRM at age 30 and RRSO at age 35	18,221	24.99	20.73	396,477	-1,995
***BRCA2*: 40% HRT adherence**					
High-risk BC surveillance and tamoxifen from age 30[b]	16,461	23.43	18.43	352,188	
RRSO at age 40 with high-risk BC surveillance and tamoxifen from age 30	14,205	24.62	19.39	373,637	-2,351
RRM at age 35	17,013	23.52	19.42	371,423	558
RRM at age 35 and RRSO at age 40	16,402	24.94	20.46	392,712	-29
***PALB2*: 40% HRT adherence**					
High-risk BC surveillance and tamoxifen from age 30[b]	10,376	23.64	18.77	365,059	
RRSO at age 45 with high-risk BC surveillance and tamoxifen from age 30	11,286	24.71	19.52	379,195	1,210
RRM at age 40	12,260	23.82	19.62	380,160	2,219
RRM at age 40 and RRSO at age 45	14,561	24.93	20.33	391,996	2,690
***RAD51C*: 40% HRT adherence**					

Strategy	Costs (£)	LYGs	QALYs	NMB[a] (£)	ICER (£/QALY)
Moderate-risk BC surveillance and tamoxifen from age 40[b]	4,947	23.68	19.59	386,873	
RRSO at age 45 with moderate-risk BC surveillance and tamoxifen from age 40	5,968	24.86	20.39	401,917	1,271
***RAD51D*: 40% HRT adherence**					
Moderate-risk BC surveillance and tamoxifen from age 40[b]	4,964	23.69	19.61	387,156	
RRSO at age 45 with moderate-risk BC surveillance and tamoxifen from age 40	5,822	24.88	20.41	402,434	1,064
***BRIP1*: 40% HRT adherence**					
No surgery[b]	1,520	23.82	20.17	401,958	
RRSO at age 45	3,767	24.99	20.91	414,493	3,040
**Overall mortality after RRSO for non-*BRCA* CSG-carriers**					
***PALB2*: No impact on overall mortality after RRSO**					
High-risk BC surveillance and tamoxifen from age 30[b]	10,376	23.64	18.77	365,059	
RRSO at age 45 with high-risk BC surveillance and tamoxifen from age 30	10,937	23.94	19.03	369,762	2,132
RRM at age 40	12,260	23.82	19.62	380,160	2,219
RRM at age 40 and RRSO at age 45	14,290	23.90	19.66	378,949	4,397
***RAD51C*: No impact on overall mortality after RRSO**					
Moderate-risk BC surveillance and tamoxifen from age 40[b]	4,947	23.68	19.59	386,873	
RRSO at age 45 with moderate-risk BC surveillance and tamoxifen from age 40	5,601	23.94	19.81	390,522	3,040
***RAD51D*: No impact on overall mortality after RRSO**					
Moderate-risk BC surveillance and tamoxifen from age 40[b]	4,964	23.69	19.61	387,156	
RRSO at age 45 with moderate-risk BC surveillance and tamoxifen from age 40	5,461	23.95	19.82	390,869	2,361
***BRIP1*: No impact on overall mortality after RRSO**					
No surgery[b]	1,520	23.82	20.17	401,958	
RRSO at age 45	3,503	23.92	20.22	400,835	46,103
***BRIP1*: No impact on overall mortality after RRSO**					
No surgery[b]	1520	23.82	20.17	401,958	

Strategy	Costs (£)	LYGs	QALYs	NMB[a] (£)	ICER (£/QALY)
RRSO at age 50	2525	23.90	20.24	402,221	15,848
**OC-risk for *BRIP1* PV-carriers**					
**RR for OC=5.54**					
No surgery[b]	2,433	23.74	20.09	399,347	
RRSO at age 45	3,716	25.03	21.00	416,239	1,412
**RR for OC=5.54 and no impact on overall mortality after RRSO**					
No surgery[b]	2,433	23.74	20.09	399,347	
RRSO at age 45	3,692	23.89	20.19	400,166	12,119
**PARP-i treatment for OC or BC**					
***BRCA1*: Olaparib for advanced OC**					
High-risk BC surveillance and tamoxifen from age 30[b]	41,333	22.86	17.76	313,844	
RRSO at age 35 with high-risk BC surveillance and tamoxifen from age 30	19,349	24.36	19.12	363,147	-16,094
RRM at age 30	42,379	23.14	19.13	340,216	763
RRM at age 30 and RRSO at age 35	19,521	25.08	20.86	397,654	-7,036
***BRCA1*: Olaparib for HER2-negative early BC and advanced OC**					
High-risk BC surveillance and tamoxifen from age 30[b]	54,019	22.97	17.84	302,851	
RRM at age 30	43,920	23.16	19.14	338,877	-7,791
RRSO at age 35 with high-risk BC surveillance and tamoxifen from age 30	32,521	24.42	19.18	350,983	-16,144
RRM at age 30 and RRSO at age 35	20,583	25.08	20.86	396,676	-11,074
***BRCA2*: Olaparib for advanced OC**					
High-risk BC surveillance and tamoxifen from age 30[b]	22,427	23.58	18.53	348,170	
RRSO at age 40 with high-risk BC surveillance and tamoxifen from age 30	14,651	24.67	19.46	374,543	-8,362
RRM at age 35	23,061	23.67	19.52	367,345	640
RRM at age 35 and RRSO at age 40	16,688	25.01	20.56	394,603	-2,820
***BRCA2*: Olaparib for HER2-negative early BC and advanced OC**					

Strategy	Costs (£)	LYGs	QALYs	NMB[a] (£)	ICER (£/QALY)
High-risk BC surveillance and tamoxifen from age 30[b]	32,980	23.66	18.59	338,779	
RRSO at age 40 with high-risk BC surveillance and tamoxifen from age 30	23,285	24.70	19.48	366,341	-10,853
RRM at age 35	25,636	23.69	19.54	365,080	-7,747
RRM at age 35 and RRSO at age 40	18,876	25.02	20.57	392,604	-7,102
***PALB2*: Olaparib plus bevacizumab for advanced OC**					
High-risk BC surveillance and tamoxifen from age 30[b]	12,303	23.66	18.78	363,385	
RRSO at age 45 with high-risk BC surveillance and tamoxifen from age 30	11,499	24.76	19.60	380,600	-980
RRM at age 40	14,189	23.84	19.63	378,482	2,221
RRM at age 40 and RRSO at age 45	14,625	25.00	20.44	394,127	1,404
***RAD51C*: Olaparib plus bevacizumab for advanced OC**					
Moderate-risk BC surveillance and tamoxifen from age 40[b]	9,945	23.73	19.62	382,537	
RRSO at age 45 with moderate-risk BC surveillance and tamoxifen from age 40	6,183	24.92	20.49	403,655	-4,335
***RAD51D*: Olaparib plus bevacizumab for advanced OC**					
Moderate-risk BC surveillance and tamoxifen from age 40[b]	10,260	23.75	19.64	382,524	
RRSO at age 45 with moderate-risk BC surveillance and tamoxifen from age 40	6,024	24.94	20.51	404,205	-4,855
***BRIP1*: Olaparib plus bevacizumab for advanced OC**					
No surgery[b]	4440	23.86	20.19	399459	
RRSO at age 45	4164	25.06	21.03	416453	-330

Note: [a]NMB was calculated using £20,000/QALY willingness-to-pay threshold; [b]Reference strategy.
Abbreviations: BC, breast cancer; CSG, cancer susceptibility gene; HER2, human epidermal growth factor receptor 2; HRT, hormone replacement therapy; ICER, incremental cost-effectiveness ratio; LYGs, life-years gained; NMB, net monetary benefit; OC, ovarian cancer; PARP-i, poly (adenosine diphosphate-ribose) polymerase inhibitor; PV, pathogenic variant; QALYs, quality-adjusted life-years; RR, relative risk; RRM, risk-reducing mastectomy; RRSO, risk-reducing salpingo-oophorectomy.

eTable-3b. Population effect of prevention and surveillance strategies per 1,000 pathogenic variant carriers for scenario analyses

Strategy	BC cases	BC deaths	OC cases	OC deaths	BC cases prevented	BC deaths prevented	OC cases prevented	OC deaths prevented
**Ages of surgery**								
***BRCA1*: older ages of surgery**								
High-risk BC surveillance and tamoxifen from age 30[a]	601	63	412	253				
RRM at age 35	162	17	419	258	439	47	-7	-4
RRSO at age 40 with high-risk BC surveillance and tamoxifen from age 30	703	59	32	12	-102	5	380	242
RRM at age 35 and RRSO at age 40	148	13	31	11	453	51	380	242
***BRCA2*: younger ages of surgery**								
High-risk BC surveillance and tamoxifen from age 30[a]	630	74	171	106				
RRSO at age 35 with high-risk BC surveillance and tamoxifen from age 30	549	33	7	2	80	41	164	103
RRM at age 30	91	15	174	107	539	59	-3	-2
RRM at age 30 and RRSO at age 35	65	5	7	2	565	69	163	103
***BRCA2*: older ages of surgery**								
High-risk BC surveillance and tamoxifen from age 30[a]	630	74	171	106				
RRM at age 40	171	18	173	107	459	56	-2	-1
RRSO at age 45 with high-risk BC surveillance and tamoxifen from age 30	568	36	10	4	62	38	161	102
RRM at age 40 and RRSO at age 45	151	10	10	4	478	64	161	102
PALB2: older ages of surgery								
High-risk BC surveillance and tamoxifen from age 30[a]	481	109	46	30				
RRSO at age 50 with high-risk BC surveillance and tamoxifen from age 30	412	44	5	2	69	64	41	27
RRM at age 40	77	18	47	30	404	91	0	0
RRM at age 40 and RRSO at age 50	61	8	5	2	420	101	41	27
***RAD51C*: older ages of surgery**								
Moderate-risk BC surveillance and tamoxifen from age 40[a]	188	53	108	66				
RRSO at age 50 with moderate-risk BC surveillance and tamoxifen from age 40	240	50	7	3	-52	4	101	63

Strategy	BC cases	BC deaths	OC cases	OC deaths	BC cases prevented	BC deaths prevented	OC cases prevented	OC deaths prevented
***RAD51D*: older ages of surgery**								
Moderate-risk BC surveillance and tamoxifen from age 40[a]	174	48	124	78				
RRSO at age 50 with moderate-risk BC surveillance and tamoxifen from age 40	222	45	8	3	-48	3	117	75
***BRIP1*: older ages of surgery**								
No surgery[a]	/	/	63	40				
RRSO at age 50	/	/	10	5	/	/	52	35
**HRT adherence following premenopausal RRSO**								
***BRCA1*: 40% HRT adherence**								
High-risk BC surveillance and tamoxifen from age 30[a]	601	63	412	253				
RRM at age 30	83	11	423	260	518	52	-11	-6
RRSO at age 35 with high-risk BC surveillance and tamoxifen from age 30	699	53	24	7	-98	10	388	246
RRM at age 30 and RRSO at age 35	64	7	24	7	537	57	387	246
***BRCA2*: 40% HRT adherence**								
High-risk BC surveillance and tamoxifen from age 30[a]	630	74	171	106				
RRSO at age 40 with high-risk BC surveillance and tamoxifen from age 30	544	32	9	3	86	42	162	103
RRM at age 35	129	16	173	107	500	57	-2	-1
RRM at age 35 and RRSO at age 40	104	7	8	3	526	67	162	103
***PALB2*: 40% HRT adherence**								
High-risk BC surveillance and tamoxifen from age 30[a]	481	109	46	30				
RRSO at age 45 with high-risk BC surveillance and tamoxifen from age 30	389	37	4	2	92	71	42	28
RRM at age 40	77	18	47	30	404	91	0	0
RRM at age 40 and RRSO at age 45	58	7	4	1	423	102	42	28
***RAD51C*: 40% HRT adherence**								
Moderate-risk BC surveillance and tamoxifen from age 40[a]	188	53	108	66				
RRSO at age 45 with moderate-risk BC surveillance and tamoxifen from age 40	228	44	6	2	-39	9	102	64

Strategy	BC cases	BC deaths	OC cases	OC deaths	BC cases prevented	BC deaths prevented	OC cases prevented	OC deaths prevented
***RAD51D*: 40% HRT adherence**								
Moderate-risk BC surveillance and tamoxifen from age 40[a]	174	48	124	78				
RRSO at age 45 with moderate-risk BC surveillance and tamoxifen from age 40	210	40	6	2	-36	8	118	76
***BRIP1*: 40% HRT adherence**								
No surgery[a]	/	/	63	40				
RRSO at age 45	/	/	7	3	/	/	56	37
**Overall mortality after RRSO for non-*BRCA* CSG-carriers**								
***PALB2*: No impact on overall mortality after RRSO**								
High-risk BC surveillance and tamoxifen from age 30[a]	481	109	46	30				
RRSO at age 45 with high-risk BC surveillance and tamoxifen from age 30	353	29	4	1	128	80	42	28
RRM at age 40	77	18	47	30	404	91	0	0
RRM at age 40 and RRSO at age 45	53	6	3	1	428	103	43	28
***RAD51C*: No impact on overall mortality after RRSO**								
Moderate-risk BC surveillance and tamoxifen from age 40[a]	188	53	108	66				
RRSO at age 45 with moderate-risk BC surveillance and tamoxifen from age 40	195	32	5	2	-7	22	102	64
***RAD51D*: No impact on overall mortality after RRSO**								
Moderate-risk BC surveillance and tamoxifen from age 40[a]	174	48	124	78				
RRSO at age 45 with moderate-risk BC surveillance and tamoxifen from age 40	180	29	6	2	-6	19	119	76
***BRIP1*: No impact on overall mortality after RRSO**								
No surgery[a]	/	/	63	40				
RRSO at age 45	/	/	7	3	/	/	56	37
***BRIP1*: No impact on overall mortality after RRSO**								
No surgery[a]	/	/	63	40				
RRSO at age 50	/	/	10	5	/	/	53	35
**OC-risk for *BRIP1* PV-carriers**								

Strategy	BC cases	BC deaths	OC cases	OC deaths	BC cases prevented	BC deaths prevented	OC cases prevented	OC deaths prevented
**RR for OC=5.54**								
No surgery[a]	/	/	99	63				
RRSO at age 45	/	/	12	5	/	/	88	58
**RR for OC=5.54 and no impact on overall mortality after RRSO**								
No surgery[a]	/	/	99	63				
RRSO at age 45	/	/	11	5	/	/	89	58
**PARP-i treatment for OC or BC**								
***BRCA1*: Olaparib for advanced OC**								
High-risk BC surveillance and tamoxifen from age 30[a]	601	63	412	171				
RRSO at age 35 with high-risk BC surveillance and tamoxifen from age 30	710	56	24	4	-108	7	388	167
RRM at age 30	83	11	423	176	518	52	-11	-4
RRM at age 30 and RRSO at age 35	65	7	25	5	536	56	387	167
***BRCA1*: Olaparib for HER2-negative early BC and advanced OC**								
High-risk BC surveillance and tamoxifen from age 30[a]	601	49	416	173				
RRM at age 30	83	9	423	176	518	40	-7	-3
RRSO at age 35 with high-risk BC surveillance and tamoxifen from age 30	710	43	24	4	-108	6	392	168
RRM at age 30 and RRSO at age 35	65	6	25	5	536	44	391	168
***BRCA2*: Olaparib for advanced OC**								
High-risk BC surveillance and tamoxifen from age 30[a]	630	74	171	72				
RRSO at age 40 with high-risk BC surveillance and tamoxifen from age 30	556	34	9	2	74	40	162	70
RRM at age 35	129	16	173	72	500	57	-2	-1
RRM at age 35 and RRSO at age 40	106	7	9	2	524	67	162	70
***BRCA2*: Olaparib for HER2-negative early BC and advanced OC**								
High-risk BC surveillance and tamoxifen from age 30[a]	630	58	172	72				
RRSO at age 40 with high-risk BC surveillance and tamoxifen from age 30	556	26	9	2	74	32	163	70

Strategy	BC cases	BC deaths	OC cases	OC deaths	BC cases prevented	BC deaths prevented	OC cases prevented	OC deaths prevented
RRM at age 35	129	13	173	73	500	45	-1	0
RRM at age 35 and RRSO at age 40	106	5	9	2	524	53	163	70
***PALB2*: Olaparib plus bevacizumab for advanced OC**								
High-risk BC surveillance and tamoxifen from age 30[a]	481	109	46	24				
RRSO at age 45 with high-risk BC surveillance and tamoxifen from age 30	402	40	4	1	79	69	42	23
RRM at age 40	77	18	47	24	404	91	0	0
RRM at age 40 and RRSO at age 45	59	7	4	1	422	102	42	23
***RAD51C*: Olaparib plus bevacizumab for advanced OC**								
Moderate-risk BC surveillance and tamoxifen from age 40[a]	188	53	108	53				
RRSO at age 45 with moderate-risk BC surveillance and tamoxifen from age 40	238	48	6	1	-50	6	102	52
***RAD51D*: Olaparib plus bevacizumab for advanced OC**								
Moderate-risk BC surveillance and tamoxifen from age 40[a]	174	48	124	63				
RRSO at age 45 with moderate-risk BC surveillance and tamoxifen from age 40	220	43	6	2	-46	5	118	61
***BRIP1*: Olaparib plus bevacizumab for advanced OC**								
No surgery[a]	/	/	63	32				
RRSO at age 45	/	/	7	2	/	/	55	30

Note: [a]Reference strategy.
Abbreviations: BC, breast cancer; CSG, cancer susceptibility gene; HER2, human epidermal growth factor receptor 2; HRT, hormone replacement therapy; OC, ovarian cancer; PARP-i, poly (adenosine diphosphate-ribose) polymerase inhibitor; PV, pathogenic variant; RR, relative risk; RRM, risk-reducing mastectomy; RRSO, risk-reducing salpingo-oophorectomy.

eReferences

1. Kuchenbaecker KB, Hopper JL, Barnes DR, et al. Risks of Breast, Ovarian, and Contralateral Breast Cancer for BRCA1 and BRCA2 Mutation Carriers. *JAMA*. 2017;317(23):2402-2416. doi:10.1001/jama.2017.7112
2. Yang X, Leslie G, Doroszuk A, et al. Cancer Risks Associated With Germline PALB2 Pathogenic Variants: An International Study of 524 Families. *J Clin Oncol*. Dec 16 2019:JCO1901907. doi:10.1200/JCO.19.01907
3. Yang X, Song H, Leslie G, et al. Ovarian and breast cancer risks associated with pathogenic variants in RAD51C and RAD51D. *J Natl Cancer Inst*. Feb 28 2020;doi:10.1093/jnci/djaa030
4. Ramus SJ, Song H, Dicks E, et al. Germline Mutations in the BRIP1, BARD1, PALB2, and NBN Genes in Women With Ovarian Cancer. *J Natl Cancer Inst*. Nov 2015;107(11)doi:10.1093/jnci/djv214
5. Cancer Research UK. *Ovarian cancer incidence statistics, 2016-2018*. 2021. Accessed 19 December 2022. https://www.cancerresearchuk.org/health-professional/cancer-statistics/statistics-by-cancer-type/ovarian-cancer/incidence#heading-One
6. Rebbeck TR, Friebel T, Lynch HT, et al. Bilateral prophylactic mastectomy reduces breast cancer risk in BRCA1 and BRCA2 mutation carriers: the PROSE Study Group. *J Clin Oncol*. Mar 15 2004;22(6):1055-62. doi:10.1200/JCO.2004.04.188
7. Finch A, Beiner M, Lubinski J, et al. Salpingo-oophorectomy and the risk of ovarian, fallopian tube, and peritoneal cancers in women with a BRCA1 or BRCA2 Mutation. *Jama*. 2006;296(2):185-192. doi:10.1001/jama.296.2.185
8. Rebbeck TR, Kauff ND, Domchek SM. Meta-analysis of risk reduction estimates associated with risk-reducing salpingo-oophorectomy in BRCA1 or BRCA2 mutation carriers. *J Natl Cancer Inst*. Jan 21 2009;101(2):80-7. doi:10.1093/jnci/djn442
9. Crosbie EJ, Flaum N, Harkness EF, et al. Specialist oncological surgery for removal of the ovaries and fallopian tubes in BRCA1 and BRCA2 pathogenic variant carriers may reduce primary peritoneal cancer risk to very low levels. *Int J Cancer*. Mar 1 2021;148(5):1155-1163. doi:10.1002/ijc.33378
10. Parker WH, Broder MS, Chang E, et al. Ovarian conservation at the time of hysterectomy and long-term health outcomes in the nurses' health study. *Obstet Gynecol*. May 2009;113(5):1027-1037. doi:10.1097/AOG.0b013e3181a11c64
11. Gaba F, Blyuss O, Tan A, et al. Breast Cancer Risk and Breast-Cancer-Specific Mortality following Risk-Reducing Salpingo-Oophorectomy in BRCA Carriers: A Systematic Review and Meta-Analysis. *Cancers*. 2023;15(5):1625. doi:10.3390/cancers15051625
12. Read MD, Edey KA, Hapeshi J, Foy C. Compliance with estrogen hormone replacement therapy after oophorectomy: a prospective study. *Menopause Int*. Jun 2010;16(2):60-4. doi:10.1258/mi.2010.010023
13. Parker WH, Feskanich D, Broder MS, et al. Long-Term Mortality Associated With Oophorectomy Compared With Ovarian Conservation in the Nurses' Health Study. *Obstetrics & Gynecology*. 2013;121(4):709-716. doi:10.1097/AOG.0b013e3182864350
14. Smith SG, Sestak I, Forster A, et al. Factors affecting uptake and adherence to breast cancer chemoprevention: a systematic review and meta-analysis. *Annals of Oncology*. 2016;27(4):575-590. doi:10.1093/annonc/mdv590
15. Cuzick J, Sestak I, Cawthorn S, Hamed H, Holli K, Howell A, Forbes JF. Tamoxifen for prevention of breast cancer: extended long-term follow-up of the IBIS-I breast cancer prevention trial. *Lancet Oncol*. Jan 2015;16(1):67-75. doi:10.1016/s1470-2045(14)71171-4

16. Cuzick J, Sestak I, Forbes JF, et al. Use of anastrozole for breast cancer prevention (IBIS-II): long-term results of a randomised controlled trial. *The Lancet*. 2020;395(10218):117-122. doi:10.1016/S0140-6736(19)32955-1
17. Warner E. Screening BRCA1 and BRCA2 Mutation Carriers for Breast Cancer. *Cancers (Basel)*. Nov 30 2018;10(12)doi:10.3390/cancers10120477
18. DiSilvestro P, Banerjee S, Colombo N, et al. Overall Survival With Maintenance Olaparib at a 7-Year Follow-Up in Patients With Newly Diagnosed Advanced Ovarian Cancer and a BRCA Mutation: The SOLO1/GOG 3004 Trial. *J Clin Oncol*. Jan 20 2023;41(3):609-617. doi:10.1200/jco.22.01549
19. Ray-Coquard I, Leary A, Pignata S, et al. Olaparib plus bevacizumab first-line maintenance in ovarian cancer: final overall survival results from the PAOLA-1/ENGOT-ov25 trial. *Ann Oncol*. Aug 2023;34(8):681-692. doi:10.1016/j.annonc.2023.05.005
20. Geyer CE, Jr., Garber JE, Gelber RD, et al. Overall survival in the OlympiA phase III trial of adjuvant olaparib in patients with germline pathogenic variants in BRCA1/2 and high-risk, early breast cancer. *Ann Oncol*. Dec 2022;33(12):1250-1268. doi:10.1016/j.annonc.2022.09.159
21. Evans DG, Howell SJ, Gandhi A, et al. Breast cancer incidence and early diagnosis in a family history risk and prevention clinic: 33-year experience in 14,311 women. *Breast Cancer Res Treat*. Oct 2021;189(3):677-687. doi:10.1007/s10549-021-06333-1
22. Wapnir IL, Anderson SJ, Mamounas EP, et al. Prognosis after ipsilateral breast tumor recurrence and locoregional recurrences in five National Surgical Adjuvant Breast and Bowel Project node-positive adjuvant breast cancer trials. *J Clin Oncol*. May 1 2006;24(13):2028-37. doi:10.1200/jco.2005.04.3273
23. Anderson SJ, Wapnir I, Dignam JJ, et al. Prognosis after ipsilateral breast tumor recurrence and locoregional recurrences in patients treated by breast-conserving therapy in five National Surgical Adjuvant Breast and Bowel Project protocols of node-negative breast cancer. *J Clin Oncol*. May 20 2009;27(15):2466-73. doi:10.1200/jco.2008.19.8424
24. Gennari A, Conte P, Rosso R, Orlandini C, Bruzzi P. Survival of metastatic breast carcinoma patients over a 20-year period: a retrospective analysis based on individual patient data from six consecutive studies. *Cancer*. Oct 15 2005;104(8):1742-50. doi:10.1002/cncr.21359
25. National Institute for Health and Care Excellence. *Early and locally advanced breast cancer: diagnosis and treatment*. National Collaborating Centre for Cancer, National Institute for Health and Care Excellence; 2009. Accessed 26 November 2022.
26. National Institute for Health and Care Excellence. *National costing report: Early and locally advanced breast cancer/Advanced breast cancer*. National Institute for Health and Care Excellence; 2009. Accessed 1 July 2023.
27. Rosenthal AN, Fraser LSM, Philpott S, et al. Evidence of Stage Shift in Women Diagnosed With Ovarian Cancer During Phase II of the United Kingdom Familial Ovarian Cancer Screening Study. *J Clin Oncol*. May 1 2017;35(13):1411-1420. doi:10.1200/jco.2016.69.9330
28. National Institute for Health and Care Excellence. *Ovarian cancer: the recognition and initial management of ovarian cancer (CG122)*. National Institute for Health and Care Excellence; 2011. Accessed 20 November 2022. https://www.nice.org.uk/guidance/cg122/resources/ovarian-cancer-recognition-and-initial-management-pdf-35109446543557

29. Office for National Statistics. *National life tables: UK*. 2021. Accessed 19 August 2023. https://www.ons.gov.uk/peoplepopulationandcommunity/birthsdeathsandmarriages/lifeexpectancies/datasets/nationallifetablesunitedkingdomreferencetables
30. Duffy S, Mackay J, Thomas S, et al. Evaluation of mammographic surveillance services in women aged 40-49 years with a moderate family history of breast cancer: a single-arm cohort study. *Health Technology Assessment (Winchester, England)*. 2013;17(11):vii-xiv, 1-95. doi:10.3310/hta17110
31. Cancer Research UK. *Ovarian cancer survival statistics*. 2022. Accessed 09 December 2022. https://www.cancerresearchuk.org/health-professional/cancer-statistics/statistics-by-cancer-type/ovarian-cancer/survival#heading-Zero
32. National Health Service. *National Cost Collection for the NHS*. 2022. Accessed 3 December 2022. https://www.england.nhs.uk/costing-in-the-nhs/national-cost-collection/#archive
33. Neuburger J, Macneill F, Jeevan R, van der Meulen JH, Cromwell DA. Trends in the use of bilateral mastectomy in England from 2002 to 2011: retrospective analysis of hospital episode statistics. *BMJ Open*. Aug 2013;3(8):e003179. doi:10.1136/bmjopen-2013-003179
34. Del Corral GA, Wes AM, Fischer JP, Serletti JM, Wu LC. Outcomes and Cost Analysis in High-Risk Patients Undergoing Simultaneous Free Flap Breast Reconstruction and Gynecologic Procedures. *Ann Plast Surg*. Nov 2015;75(5):534-8. doi:10.1097/sap.0000000000000156
35. Sun L, Brentnall A, Patel S, et al. A Cost-effectiveness Analysis of Multigene Testing for All Patients With Breast Cancer. *JAMA Oncol*. Oct 3 2019;5(12):1718-30. doi:10.1001/jamaoncol.2019.3323
36. Manchanda R, Legood R, Antoniou AC, Gordeev VS, Menon U. Specifying the ovarian cancer risk threshold of 'premenopausal risk-reducing salpingo-oophorectomy' for ovarian cancer prevention: a cost-effectiveness analysis. *J Med Genet*. Sep 2016;53(9):591-9. doi:10.1136/jmedgenet-2016-103800
37. British Heart Foundation. *Heart & Circulatory Disease Statistics*. 2022. Accessed 6 January 2023. https://www.bhf.org.uk/what-we-do/our-research/heart-statistics/heart-statistics-publications/cardiovascular-disease-statistics-2022
38. British National Formulary. *British National Formulary*. BMJ Group and Pharmaceutical Press (Royal Pharmaceutical Society of Great Britain); 2021. Accessed 3 January 2023. https://bnf.nice.org.uk/
39. Karen J, Amanda B. *Unit Costs of Health and Social Care 2021*. Personal Social Services Research Unit, University of Kent, Canterbury; 2021. Accessed 3 November 2022. https://www.pssru.ac.uk/project-pages/unit-costs/unit-costs-of-health-and-social-care-2021/
40. National Institute for Health and Care Research. *Interactive Costing Tool (iCT): Getting started*. National Institute for Health and Care Research; 2022. Accessed 22 January 2023. https://www.nihr.ac.uk/documents/interactive-costing-tool-ict-getting-started/12170
41. Gilbert L, Ramanakumar AV, Festa MC, et al. Real-world direct healthcare costs of treating recurrent high-grade serous ovarian cancer with cytotoxic chemotherapy. *Journal of Comparative Effectiveness Research*. 2020;9(8):537-551. doi:10.2217/cer-2020-0032
42. Harter P, Sehouli J, Reuss A, et al. Prospective validation study of a predictive score for operability of recurrent ovarian cancer: the Multicenter Intergroup Study DESKTOP II. A project of the AGO Kommission OVAR, AGO Study Group, NOGGO, AGO-Austria, and MITO.

International Journal of Gynecologic Cancer. 2011;21(2):289-95. doi:10.1097/IGC.0b013e31820aaafd
43. Urban RR, He H, Alfonso R, Hardesty MM, Goff BA. The end of life costs for Medicare patients with advanced ovarian cancer. *Gynecologic oncology*. 2018;148(2):336-341. doi:10.1016/j.ygyno.2017.11.022
44. National Institute for Health and Care Excellence. *Costing report: Familial breast cancer*. National Institute for Health and Care Excellence; 2013. Accessed 27 November 2022.
45. Sun L, Cromwell D, Dodwell D, et al. Costs of Early Invasive Breast Cancer in England Using National Patient-Level Data. *Value in Health*. 2020;23(10):1316-1323. doi:10.1016/j.jval.2020.05.013
46. Jeevan R, Mennie J, Mohanna P, O'Donoghue J, Rainsbury R, Cromwell D. National trends and regional variation in immediate breast reconstruction rates. *Journal of British Surgery*. 2016;103(9):1147-1156. doi:10.1002/bjs.10161
47. Miller ME, Czechura T, Martz B, et al. Operative risks associated with contralateral prophylactic mastectomy: a single institution experience. *Annals of surgical oncology*. 2013;20(13):4113-4120. doi:10.1245/s10434-013-3108-1
48. Mavaddat N, Dorling L, Carvalho S, et al. Pathology of Tumors Associated With Pathogenic Germline Variants in 9 Breast Cancer Susceptibility Genes. *JAMA Oncol*. Mar 1 2022;8(3):e216744. doi:10.1001/jamaoncol.2021.6744
49. National Institute for Health and Care Excellence. *Advanced breast cancer: diagnosis and treatment (CG81)*. National Collaborating Centre for Cancer, National Institute for Health and Care Excellence; 2009. Accessed 26 November 2022. https://www.nice.org.uk/guidance/cg81
50. National Institute for Health and Care Excellence. *Denosumab for the prevention of skeletal-related events in adults with bone metastases from solid tumours*. National Institute for Health and Care Excellence; 2012. Accessed 11 November 2022. https://www.nice.org.uk/guidance/ta265/documents/bone-metastases-from-solid-tumours-denosumab-final-appraisal-determination-guidance2
51. National Institute for Health and Care Excellence. *Early and locally advanced breast cancer: diagnosis and management (NG101)*. National Collaborating Centre for Cancer, National Institute for Health and Care Excellence; 2018. Accessed 7 November 2022. https://www.nice.org.uk/guidance/ng101
52. Coleman RE. Skeletal complications of malignancy. *Cancer: Interdisciplinary International Journal of the American Cancer Society*. 1997;80(S8):1588-1594. doi:10.1002/(sici)1097-0142(19971015)80:8+<1588::aid-cncr9>3.3.co;2-z
53. Round J, Jones L, Morris S. Estimating the cost of caring for people with cancer at the end of life: A modelling study. *Palliat Med*. Dec 2015;29(10):899-907. doi:10.1177/0269216315595203
54. Szende A, Janssen B, Cabases J. *Self-Reported Population Health: An International Perspective based on EQ-5D*. Springer; 2014. Accessed 24 January 2023.
55. Grann VR, Jacobson JS, Sundararajan V, Albert SM, Troxel AB, Neugut AI. The quality of life associated with prophylactic treatments for women with BRCA1/2 mutations. *Cancer J Sci Am*. Sep-Oct 1999;5(5):283-92.
56. Grann VR, Patel P, Bharthuar A, et al. Breast cancer-related preferences among women with and without BRCA mutations. *Breast Cancer Res Treat*. Jan 2010;119(1):177-84. doi:10.1007/s10549-009-0373-6

57. Nyman JA, Barleen NA, Dowd BE, Russell DW, Coons SJ, Sullivan PW. Quality-of-life weights for the US population: self-reported health status and priority health conditions, by demographic characteristics. *Med Care*. Jul 2007;45(7):618-28. doi:10.1097/MLR.0b013e31803dce05
58. Geuzinge HA, Obdeijn I-M, Rutgers EJ, et al. Cost-effectiveness of breast cancer screening with magnetic resonance imaging for women at familial risk. *JAMA oncology*. 2020;6(9):1381-1389. doi:10.1001/jamaoncol.2020.2922
59. De Haes J, de Koning HJ, van Oortmarssen GJ, van Agt HM, de Bruyn AE, van der Maas PJ. The impact of a breast cancer screening programme on quality‐adjusted life‐years. *International journal of cancer*. 1991;49(4):538-544. doi:10.1002/ijc.2910490411
60. Havrilesky LJ, Broadwater G, Davis DM, Nolte KC, Barnett JC, Myers ER, Kulasingam S. Determination of quality of life-related utilities for health states relevant to ovarian cancer diagnosis and treatment. *Gynecologic oncology*. 2009;113(2):216-220. doi:10.1016/j.ygyno.2008.12.026
61. Robertson C, Arcot Ragupathy SK, Boachie C, et al. The clinical effectiveness and cost-effectiveness of different surveillance mammography regimens after the treatment for primary breast cancer: systematic reviews registry database analyses and economic evaluation. *Health Technol Assess*. Sep 2011;15(34):v-vi, 1-322. doi:10.3310/hta15340
62. Cooper NJ, Abrams KR, Sutton AJ, Turner D, Lambert PC. A Bayesian approach to Markov modelling in cost-effectiveness analyses: application to taxane use in advanced breast cancer. *Journal of the Royal Statistical Society: Series A (Statistics in Society)*. 2003;166(3):389-405. doi:https://doi.org/10.1111/1467-985X.00283
63. Peasgood T, Ward SE, Brazier J. Health-state utility values in breast cancer. *Expert review of pharmacoeconomics & outcomes research*. 2010;10(5):553-566. doi:10.1586/erp.10.65
64. Heemskerk-Gerritsen BA, Seynaeve C, van Asperen CJ, et al. Breast cancer risk after salpingo-oophorectomy in healthy BRCA1/2 mutation carriers: revisiting the evidence for risk reduction. *J Natl Cancer Inst*. May 2015;107(5)doi:10.1093/jnci/djv033
65. Marcinkute R, Woodward ER, Gandhi A, et al. Uptake and efficacy of bilateral risk reducing surgery in unaffected female BRCA1 and BRCA2 carriers. *Journal of Medical Genetics*. 2022;59(2):133-140. doi:10.1136/jmedgenet-2020-107356
66. Mavaddat N, Antoniou AC, Mooij TM, et al. Risk-reducing salpingo-oophorectomy, natural menopause, and breast cancer risk: an international prospective cohort of BRCA1 and BRCA2 mutation carriers. *Breast Cancer Res*. Jan 16 2020;22(1):8. doi:10.1186/s13058-020-1247-4
67. National Institute for Health and Care Excellence. *Familial breast cancer: classification, care and managing breast cancer and related risks in people with a family history of breast cancer (CG164)*. NICE clinical guideline CG164, Updated 2023 ed. National Institute for Health and Care Excellence; 2023. Accessed 28 November 2023. https://www.nice.org.uk/guidance/cg164
68. Organisation for Economic Co-operation and Development. *Inflation (CPI)*. 2022. Accessed 10 December 2022. https://data.oecd.org/price/inflation-cpi.htm
69. Organisation for Economic Co-operation and Development. *Purchasing power parities (PPP)*. 2022. Accessed 10 December 2022. https://data.oecd.org/conversion/purchasing-power-parities-ppp.htm
70. National Institute for Health and Care Excellence. *Olaparib for maintenance treatment of BRCA mutation-positive advanced ovarian, fallopian tube or peritoneal cancer*

after response to first-line platinum-based chemotherapy. National Institute for Health and Care Excellence; 2019. Accessed 26 May 2023. https://www.nice.org.uk/guidance/ta598
71. National Institute for Health and Care Excellence. *Olaparib plus bevacizumab for maintenance treatment of advanced ovarian, fallopian tube or primary peritoneal cancer*. National Institute for Health and Care Excellence; 2021. Accessed 07 August 2023. https://www.nice.org.uk/guidance/ta693/chapter/1-Recommendations
72. National Institute for Health and Care Excellence. *Olaparib for adjuvant treatment of BRCA mutation-positive HER2-negative high-risk early breast cancer after chemotherapy*. National Institute for Health and Care Excellence; 2023. Accessed 26 May 2023. https://www.nice.org.uk/guidance/ta886/chapter/3-Committee-discussion
73. Kozlow W, Guise TA. Breast cancer metastasis to bone: mechanisms of osteolysis and implications for therapy. *Journal of mammary gland biology and neoplasia*. 2005;10(2):169-180. doi:10.1007/s10911-005-5399-8
74. McLaughlin JR, Rosen B, Moody J, et al. Long-term ovarian cancer survival associated with mutation in BRCA1 or BRCA2. *Journal of the National Cancer Institute*. 2013;105(2):141-148. doi:10.1093/jnci/djs494
75. Nahshon C, Barnett-Griness O, Segev Y, Schmidt M, Ostrovsky L, Lavie O. Five-year survival decreases over time in patients with BRCA-mutated ovarian cancer: a systemic review and meta-analysis. *Int J Gynecol Cancer*. Jan 2022;32(1):48-54. doi:10.1136/ijgc-2020-001392
76. Kotsopoulos J, Rosen B, Fan I, et al. Ten-year survival after epithelial ovarian cancer is not associated with BRCA mutation status. *Gynecol Oncol*. Jan 2016;140(1):42-7. doi:10.1016/j.ygyno.2015.11.009
77. Yao Q, Liu Y, Zhang L, et al. Mutation Landscape of Homologous Recombination Repair Genes in Epithelial Ovarian Cancer in China and Its Relationship With Clinicopathlological Characteristics. *Front Oncol*. 2022;12:709645. doi:10.3389/fonc.2022.709645
78. Finch AP, Lubinski J, Møller P, et al. Impact of oophorectomy on cancer incidence and mortality in women with a BRCA1 or BRCA2 mutation. *J Clin Oncol*. May 20 2014;32(15):1547-53. doi:10.1200/jco.2013.53.2820
79. Domchek SM, Friebel TM, Singer CF, et al. Association of risk-reducing surgery in BRCA1 or BRCA2 mutation carriers with cancer risk and mortality. *Jama*. Sep 1 2010;304(9):967-75. doi:10.1001/jama.2010.1237
80. McCarthy AM, Menke A, Ouyang P, Visvanathan K. Bilateral Oophorectomy, Body Mass Index, and Mortality in U.S. Women Aged 40 Years and Older. *Cancer Prevention Research*. 2012;5(6):847-854. doi:10.1158/1940-6207.Capr-11-0430
81. Parker WH. Bilateral oophorectomy versus ovarian conservation: effects on long-term women's health. *J Minim Invasive Gynecol*. Mar-Apr 2010;17(2):161-6. doi:10.1016/j.jmig.2009.12.016
82. Rocca WA, Grossardt BR, de Andrade M, Malkasian GD, Melton LJ, 3rd. Survival patterns after oophorectomy in premenopausal women: a population-based cohort study. *Lancet Oncol*. Oct 2006;7(10):821-8. doi:10.1016/s1470-2045(06)70869-5
83. Wilson LF, Pandeya N, Byles J, Mishra GD. Hysterectomy status and all-cause mortality in a 21-year Australian population-based cohort study. *Am J Obstet Gynecol*. Jan 2019;220(1):83.e1-83.e11. doi:10.1016/j.ajog.2018.10.002

84. Duan L, Xu X, Koebnick C, et al. Bilateral oophorectomy is not associated with increased mortality: the California Teachers Study. *Fertil Steril*. Jan 2012;97(1):111-7. doi:10.1016/j.fertnstert.2011.10.004
85. National Institute for Health and Care Excellence. *NICE health technology evaluations: the manual*. Process and methods [PMG36] ed. National Institute for Health and Care Excellence; 2022. Accessed 19 October 2022. https://www.nice.org.uk/process/pmg36/chapter/introduction-to-health-technology-evaluation
86. Drummond MF, Sculpher MJ, Claxton K, Stoddart GL, Torrance GW. *Methods for the economic evaluation of health care programmes*. Oxford university press; 2015.
87. Ara R, Wailoo A. Using Health State Utility Values in Models Exploring the Cost-Effectiveness of Health Technologies. *Value in Health*. 2012/09/01/ 2012;15(6):971-974. doi:https://doi.org/10.1016/j.jval.2012.05.003
