# Supplementary material for: Cost-Effectiveness of Gene-Specific Prevention Strategies for Ovarian and Breast Cancer
Source: JAMA Netw Open. 2024 Feb 9;7(2):e2355324. doi: 10.1001/jamanetworkopen.2023.55324 (PMC10858404; doi:10.1001/jamanetworkopen.2023.55324)
Supplement: Supplement 2. — Data Sharing Statement [file jamanetwopen-e2355324-s002.pdf]

Data Sharing Statement

Wei. Cost-Effectiveness of Gene-Specific Prevention Strategies for Ovarian and Breast Cancer. *JAMA Netw Open*. Published February 09, 2024.
doi:10.1001/jamanetworkopen.2023.55324

Data
Data available: Yes
Data types: Other (please specify)
Additional Information: Analysis has used published and available data. This can be made available from a reasonable request to the corresponding author- Ranjit Manchanda , email- r.manchanda@qmul.ac.uk
How to access data: Analysis has used published and available data. This can be made available from a reasonable request to the corresponding author- Ranjit Manchanda , email- r.manchanda@qmul.ac.uk
When available: With publication

Supporting Documents
Document types: None

Additional Information
Who can access the data: Researchers whose proposed use of the data has been approved
Types of analyses: Specified purpose
Mechanisms of data availability: after approval of a proposal, with a signed data access agreement
